# Structural and Biochemical Insights Into Two BAHD Acyltransferases (*At*SHT and *At*SDT) Involved in Phenolamide Biosynthesis

**DOI:** 10.3389/fpls.2020.610118

**Published:** 2021-01-13

**Authors:** Chengyuan Wang, Jianxu Li, Miaolian Ma, Zhaozhu Lin, Wenli Hu, Wei Lin, Peng Zhang

**Affiliations:** ^1^National Key Laboratory of Plant Molecular Genetics, Center for Excellence in Molecular Plant Sciences, Shanghai Institute of Plant Physiology and Ecology, Chinese Academy of Sciences, Shanghai, China; ^2^Department of Microbiology and Immunology, School of Medicine & Holistic Integrative Medicine, Nanjing University of Chinese Medicine, Nanjing, China

**Keywords:** BAHD family acyltransferases, spermidine, acyl acceptor, phenolamides, multisite-acylation, crystal structure

## Abstract

Phenolamides represent one of the largest classes of plant-specialized secondary metabolites and function in diverse physiological processes, including defense responses and development. The biosynthesis of phenolamides requires the BAHD-family acyltransferases, which transfer acyl-groups from different acyl-donors specifically to amines, the acyl-group acceptors. However, the mechanisms of substrate specificity and multisite-acylation of the BAHD-family acyltransferases remain poorly understood. In this study, we provide a structural and biochemical analysis of *At*SHT and *At*SDT, two representative BAHD-family members that catalyze the multisite acylation of spermidine but show different product profiles. By determining the structures of *At*SHT and *At*SDT and using structure-based mutagenesis, we identified the residues important for substrate recognition in *At*SHT and *At*SDT and hypothesized that the acyl acceptor spermidine might adopt a free-rotating conformation in *At*SHT, which can undergo mono-, di-, or tri-acylation; while the spermidine molecule in *At*SDT might adopt a linear conformation, which only allows mono- or di-acylation to take place. In addition, through sequence similarity network (SSN) and structural modeling analysis, we successfully predicted and verified the functions of two uncharacterized *Arabidopsis* BAHD acyltransferases, OAO95042.1 and NP_190301.2, which use putrescine as the main acyl-acceptor. Our work provides not only an excellent starting point for understanding multisite acylation in BAHD-family enzymes, but also a feasible methodology for predicting possible acyl acceptor specificity of uncharacterized BAHD-family acyltransferases.

## Introduction

Phenolamides, also known as phenylamides or hydroxycinnamic acid amides, are ubiquitous secondary metabolites in plants. They are produced by mono-conjugation of aromatic monoamines (tyramine, tryptamine, and dopamine) or poly-conjugation of aliphatic polyamines (putrescine, spermidine, spermine, and agmatine) with phenolic acids (coumaric, caffeic, ferulic, and sinapoyl acids; Edreva et al., 2007). Numerous studies have suggested that phenolamides play critical roles in plant biotic or abiotic stress responses (Keller et al., 1996; Von Ropenack et al., 1998; Walters et al., 2001; Galis et al., 2006; Edreva et al., 2007; Muroi et al., 2009; Kaur et al., 2010; Park et al., 2014; Cho and Lee, 2015; Schafer et al., 2017). The accumulation of *p*-coumaroyl/feruloyl-agmatine and *p*-coumaroyl/feruloyl-putrescine was induced in leaves by pathogenic infections of *Solanum tuberosum* and *Arabidopsis thaliana* (Keller et al., 1996; Von Ropenack et al., 1998; Muroi et al., 2009). Two major phenolamides of *Nicotinana attenuate* plants, caffeoylputrescine and dicaffeoylspermidine, increased dramatically in local and systemic tissues after an herbivore attack (Keller et al., 1996; Galis et al., 2006; Schafer et al., 2017). N-*trans*-cinnamoyltryptamine and N-*p*-coumaroylserotonin could be isolated from UV-treated rice leaves, and the genes for arylamine biosynthesis of them were also up-regulated by UV irradiation (Park et al., 2014).

In recent years, enzymes and genes accounting for the biosynthesis of mono- or di- substituted phenolamides in plants have been identified through biochemical and metabolome-based genome-wide association study (GWAS) approaches, and their expression has been analyzed (Grienenberger et al., 2009; Luo et al., 2009; Onkokesung et al., 2012; Wen et al., 2014; Dong et al., 2015; Elejalde-Palmett et al., 2015; Delporte et al., 2018). Agmatine coumaroyltransferase (ACT) synthesizes *p-*coumaroyl/feruloyl-agmatine and *p-*coumaroyl/feruloyl-putrescine in the *Arabidopsis* leaves and barley seedlings (Burhenne et al., 2003; Muroi et al., 2009). Spermidine disinapoyl/dicoumaroyl transferases *At*SDT and *At*SCT were expressed mainly in the seed and root of *Arabidopsis* (Luo et al., 2009). Putrescine hydroxycinnamoyl transferase (AT1) and spermidine hydroxycinnamoyl acyltransferase (DH29) were found to be expressed in the leaves of native tobacco (Onkokesung et al., 2012). Two spermidine hydroxycinnamoyl transferases were identified in leaves, while four putrescine hydroxycinnamoyl transferases were found to be constitutively expressed in *Oryza sativa* flowers, roots, and leaves (Wen et al., 2014; Tanabe et al., 2016). In contrast to the mono- or di- substituted phenolamides, which seem to be widely distributed in different organs, the poly-substituted phenolamides, tri-substituted spermidine, and tetra-substituted spermidine were found to be restricted to the flower pollen coat. Spermidine/spermine hydroxycinnamoyl transferases (SHT), accounting for the synthesis of tri- or tetra- substituted spermidine/spermidine, have been identified and studied in *Arabidopsis thaliana* (*At*SHT)*, Malus domestica* (*Md*SHT), *and Cichorium intybus* (*Ci*SHT; Grienenberger et al., 2009; Elejalde-Palmett et al., 2015; Delporte et al., 2018).

All these phenolamide synthesis enzymes belong to the acyl-coenzyme A (CoA)-dependent BAHD acyltransferases family, which was named according to the first letter of each of its first four biochemically characterized enzymes: Benzylalcohol O-acetyltransferase, Anthocyanin O-HCT, HCT of anthranilate, and Deacetylvindoline 4-O-acetyltransferase (D’Auria, 2006). It has been established that BAHD acyltransferases catalyze the transfer of acyl groups from acyl-donors (Acyl-CoA) to various acyl-acceptors. Several structural studies of BAHD acyltransferases have been also reported (Ma et al., 2005; Unno et al., 2007; Garvey et al., 2008, 2009; Lallemand et al., 2012; Chiang et al., 2018; Levsh et al., 2019). However, the BAHD family enzymes in plants have complicated substrates (various acyl-donors and acceptors) and diverse products, while only sharing low sequence similarities, which makes it extremely challenging to predict the possible substrate specificity of uncharacterized BAHD family enzymes from their amino acid sequences. In addition, the multisite acylation mechanisms of BAHD-family acyltransferases also remain poorly understood until now.

In this study, we first performed structural analyses on two representative BAHD family enzymes, the *Arabidopsis* mono-/di-acyltransferase-SDT (*At*SDT) and poly acyltransferase-SHT (*At*SHT), then explored the acceptor-molecule specificity and potential molecular mechanisms of multisite acylation by structure-based mutagenesis approaches, and, finally, successfully predicted and varified the acyl acceptor substrates of two uncharacterized BAHD family transferases in *Arabidopsis* using our prediction methods on the basis of a sequence similarity network (SSN) and of structure modeling. All these results here could not only could deepen our understanding of substrate specificity and multisite acylation mechanisms of this large enzyme family, but also provide important insights on the function of many diverse and so far, uncharacterized BAHD-family proteins in plants.

## Materials and Methods

### Gene Cloning, Site-Directed Mutagenesis, Expression, and Protein Purification

Wild type *SDT* (*AT2G19070*), *SHT* (*AT2G23510*), OAO95042.1 (*AT5G07080*), and NP_190301.2 (*AT3G47170*) genes from *Arabidopsis thaliana* were cloned into the pET28a vector under control of the bacteriophage T7 gene promoters using NdeI and XhoI. The resulting plasmids were transformed into *Escherichia coli* strain BL21(DE3; Invitrogen). Single colonies of the resulting transformants were used to inoculate 50 ml LB broth containing 50 μg/ml kanamycin, and cultures were incubated 16 h at 37°C with shaking. Aliquots (10 ml) were used to inoculate 1 L LB broth containing 50 μg/ml kanamycin, cultures were incubated at 37°C with shaking until OD_600_ = 0.8, cultures were induced by addition of isopropyl-β-D-thiogalactoside to 1 mM, and cultures were incubated 16 h at 16°C. Cells were harvested by centrifugation (4,000 × *g*; 15 min at 4°C), re-suspended in buffer A (10 mM Tris-HCl, pH 8.0, 200 mM NaCl, 5 mM DTT, and 5% glycerol), and were lysed using an EmulsiFlex-C5 cell disruptor (Avestin). The lysate was centrifuged (20,000 × *g*; 30 min at 4°C) and the cell debris was removed. The supernatant was loaded onto a 5 ml column of Ni^2+^-NTA-agarose (Qiagen) pre-equilibrated in buffer A, and the column was washed with 10 × 5 ml buffer A containing 25 mM imidazole and eluted with 50 ml buffer A containing 250 mM imidazole. The sample was concentrated to around 10.0 mg/ml and further purified by gel filtration chromatography on a HiLoad 16/60 Superdex 200 prep grade column (GE Healthcare) in 20 mM Tris-HCl, pH 8.0, 100 mM NaCl, 5 mM MgCl_2_, and 1 mM β-mercaptoethanol; The peak fractions were collected and concentrated to 10 mg/ml in the same buffer using 30 kDa MWCO Amicon Ultra-15 centrifugal ultrafilters (EMD Millipore); and stored in aliquots at −80°C. Yields were ~5 mg/l, and purities were ~95%. Site-directed mutations were prepared using one step PCR method, and proteins were expressed and purified following similar protocols as wild type.

### *In vitro* Activity Assays

The *in vitro* activity assays of the purified recombinant protein were performed at 30°C for 30 min in 100 μl 100 mM Tris-HCl buffer (pH 7.5) containing 60 μM acyl donor (caffeoyl-CoA or feruloyl-CoA or sinapoyl-CoA) and 200 μM acyl acceptor (spermidine or spermine or putrescine) and 10 μg purified protein. The reactions were terminated by adding 20 μl ice-cold 0.5% trifluoroacetic acid and directly subjected to liquid chromatography-mass spectrometry (LC-MS) on an Agilent 1260 system (Agilent Technologies, CA), equipped with electron spray ionization mass spectrometer 6125B. The temperature of column oven is 30°C; Electron Spray Ionization (ESI) is in the positive mode; capillary voltage is at 3 Kv; and for full-scan mode, the wavelength range is from 190 to 600 nm. Samples were separated on a reverse-phase C18 column [Thermo Syncronis C18 analytical column (150 mm × 4.6 mm, 5 μm)] at a flow rate of 0.8 ml/min and a gradient mobile phase as follows: 0–5 min, 15% solvent B (0.2% acetic acid in acetonitrile) in solvent A (0.2% acetic acid in water); 5–25 min, 15–100% solvent B; 25–35 min, 100% solvent B; 35–40 min, 100 to 15% B (Grienenberger et al., 2009; Luo et al., 2009). CoA esters were synthesized according to published methods (Semler et al., 1987) and were identified and quantified by spectrophotometry (Stockigt and Zenk, 1974). All of the reactions were run for two technical replicates, and each assay was repeated for at least three independent experiments.

### Crystallization, Data Collection and Structure Determination

Robotic crystallization trials were performed for *At*SDT co-crystallized with spermidine and CoA-HS for *At*SHT co-crystallized with spermdine and CoA-HS by using a Gryphon liquid handling system (Art Robbins Instruments), commercial screening solutions (Emerald Biosystems, Hampton Research, and Qiagen), and the sitting-drop vapor-diffusion technique (drop: 0.2 μl protein plus 0.2 μl screening solution; reservoir: 60 μl screening solution; 20°C). About 900 conditions were screened. Under several conditions, *At*SDT and *At*SHT crystals appeared within 2 weeks. Conditions were optimized using the hanging-drop vapor-diffusion technique at 20°C. The optimized crystallization condition for *At*SDT was 0.1 M MES (pH 6.5), 25% W/V PEG 4000 at 20°C; the optimized crystallization condition for AtSHT was 0.2 M ammonium sulfate, 0.1 M Tris-HCl (pH 8.5), 25% W/V PEG 3350 at 20°C. Crystals were transferred to a reservoir solution containing 20% (v/v) glycerol and flash-cooled with liquid nitrogen.

Diffraction data were collected from cryo-cooled crystals at SSRF BL17U. Data were processed using HKL2000 (Otwinowski and Minor, 1997). The resolution cut-off criteria were: (i) I/σ < =2.0 and (ii) CC_1/2_ (highest resolution shell) < 0.5.

The structures of *At*SDT and *At*SHT were solved by molecular replacement with MOLREP (Collaborative Computational Project Number 4, 1994) using the structure of native HCT from *Coffea canephora* (PDB 4G0B) as a starting model. The molecular replacement solution was outstanding, and an automatic model building was performed with Phenix (Adams et al., 2010). Additional model building was done manually with Coot (Emsley et al., 2010) and refined with Phenix. The final model of *At*SDT and *At*SHT was refined to 2.4 Å and 2.3 Å resolution, respectively. The final models for *At*SDT and *At*SHT were refined to R_work_ and R_free_ of 0.19/0.24 and 0.19/0.25, respectively (Table 1).

**Table 1:** Structure data collection and refinement statistics.

Complex	*At*SDT∙spermidine	*At*SHT∙spermidine∙CoA
Data collection source	SSRL BL17U	SSRL BL17U
PDB code	6LPW	6LPV
**Data collection**		
Space group	P2_1_2_1_2_1_	P2_1_2_1_2_1_
Cell dimensions		
a, b, c (Å)	60.989,103.062,103.062	57.364,90.433,95.248
α, β, γ (°)	90.0, 90.0, 90.0	90.0, 90.0, 90.0
Resolution (Å)	48.16–2.40 (2.49–2.40)	49.1–2.29 (2.37–2.29)
Number of unique reflections	32,701	22,640
R_merge_	0.064 (0.469)	0.076 (0.428)
R_meas_	0.126 (0.806)	0.119 (0.803)
R_pim_	0.045 (0.280)	0.045 (0.301)
CC_1/2_(highest shell)	0.871	0.793
I/σI	15.47 (5.45)	15.94 (4.53)
Completeness (%)	95.82 (90.88)	99.43 (95.63)
**Refinement**		
Number of unique reflections	32,701	22,640
Number of test reflections	3,029	2,144
R_work_/R_free_	0.19/0.24 (0.23/0.26)	0.19/0.25 (0.23–0.24)
Number of atoms		
Protein	6,786	3,662
Ligand/ion Water	29 102	80
r.m.s.deviations		
Bond lengths (Å)	0.014	0.026
Bond angles (°)	1.41	1.64
MolProbity statistics		
Clashscore	7.57	9.23
Rotamer outliers (%)	0	1.4
Cβ outliers (%)	0	0
Ramachandran plot		
Favored (%)	98	97
Outliers (%)	0	0.23

Highest resolution shell in parentheses.

### Molecular Docking Studies

All molecular docking studies were performed using Autodock4.2 package (Morris et al., 2009). Briefly, the crystal structure of *At*SHT or *At*SDT was docked with their potential products (CoA-HS for *At*SHT/*At*SDT when docking with their final products or intermediate products). The molecule was added with non-polar hydrogens and assigned partial atomic charges using AutoDockTools (ADT; Morris et al., 2009). The coordinates of feruloyl-CoA and spermidine in *At*SHT structure were generated based on the coordinates of *p*-coumaroyl shikimate from the crystal structure of *At*HCT (PDB 5KJT) and of HS-CoA from the crystal structure of *At*SHT (PDB 6LPV) in combination with the CORINA Classic online service. A grid box with 40 × 40 × 40 grid points and 0.2 Å grid spacing centered roughly at the feruloyl-CoA binding position was used as the searching space. 100 runs of Larmarckian Genetic Algorithm were performed to search the protein-ligand interactions. The results were clustered and ranked. Result analyses and figure rendering were performed using PyMOL.

### Sequence Similarity Network and Phylogenetic Analysis

The sequence datasets of the BAHD family were gathered by using *At*SDT, *At*SHT, and *At*HCT as searching the template for Blast e-value cut-off at 1 × 10^−25^. All the sequences were filtered by a redundancy check and a conserve motif search. The final number of 12,768 non-redundant BAHD proteins were further used to generate the sequence similarity network (SSN) by using Pythoscape (Barber and Babbitt, 2012) and visualized at e-value cut-off 1 × 10^–51.5^ in Cytoscape. Two hundred and twenty-seven protein sequences from each of the clusters in the SSN, including *At*SCT, *At*SHT, *At*HCT, *At*SDT, were used to generate the neighbor-joining tree by MEGA8 software (Tamura et al., 2013) and draw the final maps using the iTOL online software.

### Data Availability

The crystal structures of *At*SHT and *At*SDT have been deposited into Protein Data Bank under accession numbers 6LPV and 6LPW. GenBank accession numbers: *AT5G07080* for OAO95042.1 and *AT3G47170* for NP_190301.2.

## Results

### Structural and Biochemical Characterization of *At*SHT Enzyme

**Figure 1:**
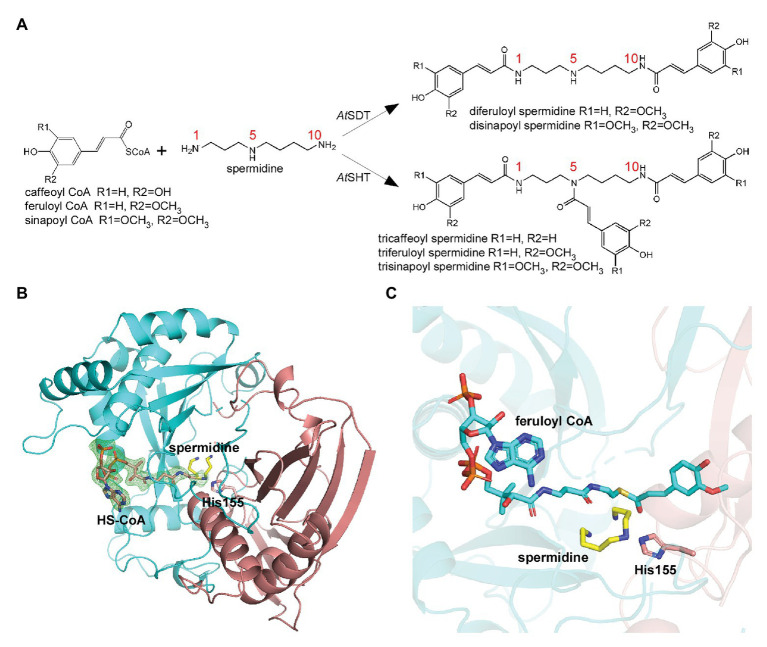
Overall structure of *At*SHT. **(A)** Spermidine conjugates by *At*SHT and *At*SDT in *Arabidopsis*. *At*SDT catalyzes the di- acylation of spermidine N^1^ and N^10^, while *At*SHT catalyzes the tri- acylation of spermidine N^1^, N^5^, and N^10^. **(B)** Overall structure of *At*SHT bound with spermidine and HS-CoA. Residues 1–201 are colored red and residues 202–451 are colored cyan. Spermidine and HS-CoA are shown with yellow and pink stick models, respectively. The conserved catalytic residue His155 is also shown with sticks; the Fo-Fc electron density omits the map for HS-CoA (contoured at 2.5σ), is shown with green mesh. **(C)** The modeled structure of *At*SHT showing the binding site of feruloyl-CoA and spermidine. The feruloyl CoA is shown with cyan sticks.

BAHD family acyltransferases, *At*SHT and *At*SDT, both catalyze the spermidine acylation. *At*SHT uses hydroxycinnamoyl CoAs, including caffeoyl/feruoyl/p-coumaroyl/sinapoyl-CoA as acyl donors to fully substitute the N^1^, N^5^, and N^10^ positions of spermidine (Grienenberger et al., 2009), while *At*SDT uses caffeoyl/feruoyl/sinapoyl-CoA with the N^1^ and N^10^ positions of spermidine (Figure 1A; Supplementary Figure 1; Luo et al., 2009). Despite the similar substrates’ specificity, they differ greatly in sequence identity (only sharing 22.2%) and the final products’ acyl-group substitution sites (Supplementary Figures 2–4). In addition, the expression pattern and distribution of these two enzymes in *Arabidopsis thaliana* are different (Grienenberger et al., 2009; Luo et al., 2009; Dong et al., 2015; Elejalde-Palmett et al., 2015; Delporte et al., 2018). All of this suggests that *At*SHT and *At*SDT may gain similar enzyme activities through a possible convergent-evolutionary pathway. To elucidate the molecular basis underlying differences in the acceptor-molecule specificity and possible molecular multisite acylation mechanisms of *At*SHT and *At*SDT, we expressed the *At*SHT and *At*SDT enzymes, and determined the crystal structure of *At*SHT in complex CoA-HS at 2.2 Å resolution, as well as the crystal structure of *At*SDT in the apo form at 2.4 Å resolution. Statistics of data collection and model refinement are summarized in Table 1. The overall structures of both *At*SHT and *At*SDT are similar to known BAHD family acyltransferases. The structures consist of two pseudo-symmetric N-terminal and C-terminal domains that are connected by a long loop. The N-terminal (residues 1–173 and 391–411) and C-terminal (residues 230–390 and 412–451) domains of *At*SHT feature a similar spatial arrangement with a β-sheet core flanked by α-helices (Figure 1B). The active site is located at the interface of the two domains with a residue His155 in between. The extra density near His155 was determined as CoA-HS (Figure 1B). The 3'-phosphoadenosine group of CoA-HS binds with residues Arg246, Arg263, Ser387, and Thr390 in the structure, while residues Glu265, Thr262, and Arg298 interact with the diphosphate group of CoA-HS (Supplementary Figure 5), and these residues are conserved in both *At*SDT and *At*SHT (Supplementary Figure 4). To view the relative positions of the acyl acceptor and donor, we modeled the potential acyl donor feruloyl-CoA and the acyl-acceptor spermidine into the *At*SHT structure by using *At*HCT/p-coumaroyl-CoA (PDB ID 5KJT) and *At*HCT/p-coumaroylshikimate (PDB ID 5KJU) structures as references (Figure 1C).

**Figure 2:**
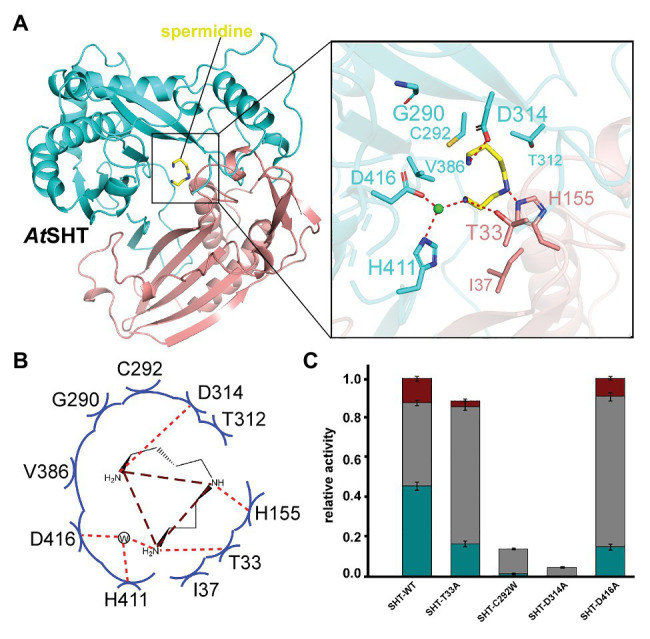
The acyl acceptor-spermidine-binding site of *At*SHT. **(A)** Overall structure of *At*SHT bound with spermidine. Zoom-in view shows the residues interacting with spermidine. Color coding is the same as in Figure 1B. The light green sphere indicates a water molecule. **(B)** Summary of residue interactions with spermidine. Red dashed lines indicate H-bonds; blue arcs indicate van der Waals interactions. **(C)** A histogram showing the relative activity of wide-type and mutant *At*SHTs. The mono-, di-, and tri- acylated products are shown with dark-cyan, gray, and dark-red colors, respectively. Values are means ± S.D., and error bars indicate the S.D. for three biological replicates.

In the modeled structure, several hydrophobic residues were found to surround the feruloyl head, which could serve to improve the affinity-to-fit the phenolic acid group and is consistent with the previous study (Levsh et al., 2016; Supplementary Figure 6). Interestingly, we observed a possible electron density in the modeled position of spermidine from the *At*SHT map. The residues around the position form a C-shape channel, which is highly negative charged (Figures 2A,B). We further used spermidine and N^1^, N^5^,N^10^-trihydroxyferuloyl spermidine as ligands to dock in the position, and the results shows both of them fit well in the channel (Supplementary Figure 7). Based on our docking results, spermidine seems to be able to adopt three differently rational conformations that each fits the electron density, and interactions with the surrounding protein residues make a head-to-tail “C” shape (Figures 2A,B). In all three possible conformations, spermidine can form hydrogen bonds with Asp314, Thr33, and the conserved catalytic residue His155. In addition, residues Asp416 and His411, as well as a water molecule, form a hydrogen-bonding network stabilizing spermidine; while residues Cys292, Gly290, Thr312, Val386, and Ile37 further stabilize spermidine *via* van der Waals interactions (Figures 2A,B); all of these interaction residues form a possible spermidine binding pocket in *At*SHT.

To verify this potential spermidine binding pocket in the *At*SHT structure, we mutated the relevant residues within the spermidine binding pockets of *At*SHT to assess the effects on enzyme activity and reaction products. The experimental results clearly showed that wild-type *At*SHT can efficiently convert spermidine and feruloyl-CoA to monoferuloyl spermidine (46%), diferuloyl spermidine (42%), and triferuloyl spermidine (12%; Figure 2C; Supplementary Figure 2A). *At*SHT mutation D314A absolutely abolishes the hydrogen-bonding interaction with the amino group of spermidine, and mutation C292W disrupts the binding of spermidine, thereby resulting in substantially decreased *At*SHT enzymatic activity (Figure 2C). In addition, the T33A mutation destabilizes the binding of spermidine and impairs the enzyme activity (fully substituted spermidine was reduced from 12 to 3%), while D416A had less effect on the enzymes as it interacts with spermidine *via* a water molecule. These results confirmed the potential interactions within the proposed spermidine binding pocket of the *At*SHT crystal structure (Figure 2).

### Structural and Biochemical Characterization of *At*SDT Enzyme

**Figure 3:**
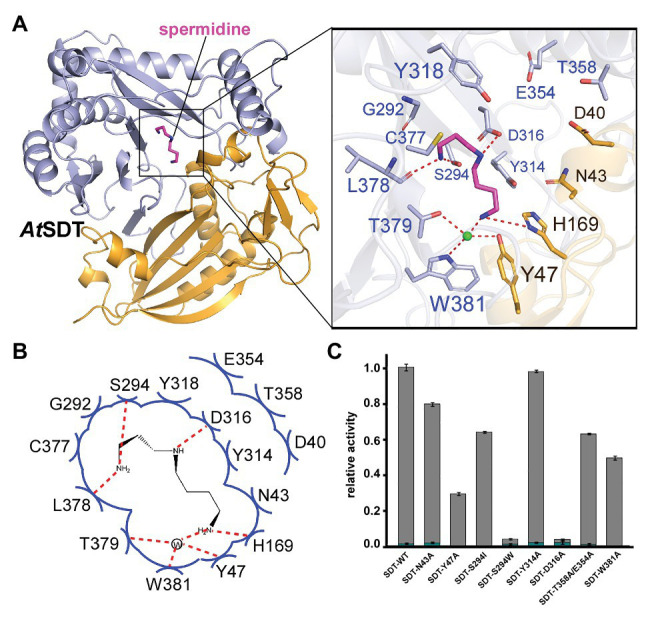
The acyl acceptor spermidine-binding site of *At*SDT. **(A)** Overall structure of *At*SDT bound with spermidine. Residues 1–200 are colored light orange, residues 201–449 are colored light blue; spermidine is shown as a magenta stick model. Zoom-in view shows the residues interacting with spermidine. The light green sphere indicates a water molecule. **(B)** Summary of residue interactions with spermidine. Red dashed lines indicate H-bonds; blue arcs indicate van der Waals interactions. **(C)** A histogram showing the relative activity of wide-type and mutant *At*SDTs. The mono- and di- acylated products are shown with dark cyan and gray colors, respectively. Values are means ± S.D., and error bars indicate the S.D. for three biological replicates.

*At*SDT adopts an overall structure that is similar to *At*SHT (RMSD = 1.68 Å), which also contains two pseudo-symmetric N-terminal (residues 1–187 and 384–407) and C-terminal (residues 233–383 and 408–451) domains that are connected by a long loop (residues 188–232; Figure 3A). No extra electron density for the acyl donor or the reaction product CoA-HS was seen in the *At*SDT map (in contrast with *At*SHT), which may be due to the crystal packing since the crystallography symmetry related *At*SDT molecule blocks the entry binding tunnel, thereby preventing acyl donor and putative reaction product entry. Unexpectedly, after superimposed with the *At*SHT structure, we observed a possible electron density for spermidine in the corresponding modeled acyl-acceptor position from the *At*SDT map. The residues around the position form a linear-shape channel, which is highly negative charged (Figures 3A,B). We further used spermidine, N^1^,N^10^-dihydroxysinapoyl spermidine, N^1^,N^5^,N^10^-trihydroxysinapoyl spermidine as ligands to dock in the position and the results show spermidine and N^1^,N^10^-dihydroxysinapoyl spermdine fit well in the channel but crash with N^1^,N^5^,N^10^-trihydroxysinapoyl spermidine (Supplementary Figures 7B,C). According to the docking results, the spermidine in *At*SDT seems be able to adopt a linear rational conformation to fit that electron density and forms a hydrogen bond through its τ-nitrogen with the conserved catalytic residue His169. This spatial arrangement is consistent with the role of His169 as a general base that deprotonates the acyl-acceptor spermidine, priming it for the nucleophilic attack of the carbonyl carbon of the hydroxycinnamoyl-CoA acyl donor. Specificity-determining residues, Asp316 and Ser294, form hydrogen bonds with the N^1^ and N^5^ groups of spermidine. In addition, Tyr47, Trp381, and Thr379 form hydrogen bonds through a water molecule with the spermidine N^10^ group. These interactions stabilize the binding of spermidine. Furthermore, N-terminal domain residues Tyr314, Tyr318, Cys377, Glu354, Thr358, and Gly292, and C-terminal domain residues, Asp40 and Asn43, contact spermidine *via* van der Waals interactions (Figures 3A,B). All of these surrounding residues will also form a possible spermidine binding pocket in *At*SDT, and spermidine may adopt a totally different conformation from that in *At*SHT (Figures 3A,B).

To confirm this potential spermidine binding pocket in the *At*SDT structure, we mutated the relevant residues within the spermidine-binding pockets of *At*SDT to assess the effects on enzyme activity and reaction products. The results showed that wild-type *At*SDT can efficiently couple spermidine with sinapoyl CoA to yield monosinapoyl (2%) spermidine and disinapoyl spermidine (98%; Figure 3C; Supplementary Figure 3). Meanwhile, eight single or double *At*SDT mutants that potentially affect the binding of spermidine were generated. The results showed that the D316A mutation absolutely abolishes the hydrogen bonding interaction with the N5 group of spermidine, and mutant S294W not only disrupts the potential hydrogen bonding interaction with the N1 group, but also prevents spermidine binding, thereby substantially decreasing *At*SDT enzymatic activities. The S294I mutation impairs the enzyme activity (compared with wild-type *At*SDT, the production of disinapoyl spermidine was reduced from 98 to 63%), suggesting that disruption of the hydrogen bond interaction destabilizes spermidine binding. In addition, the Y47A and W381A mutations seem to have a greater effect on the enzyme activity than the S294I mutation (the product disinapoyl spermidine was reduced from 98 to 28% or 49%, respectively), as these two residues also form the hydrogen bond interactions with spermidine *via* a water molecule, and these results indicated that the hydrogen bond interaction between S294 has less effect on the enzyme activity than that of Y47 or W384 and spermidine, while the N43A and Y314A mutations disrupt the van der Waals interactions with spermidine, slightly impairing enzyme activities (Figure 3C). Furthermore, the double mutant E354A/T358A, which changes residues located at the predicted spermidine entry channel in the *At*SDT structure (Supplementary Figure 8A), showed decreased activity compared with wild-type *At*SDT (Figure 3C). Interestingly, these two residues are also close to a loop (Met364-Leu375 in *At*SDT) which is unstable compared with other parts of the structure and shows a huge movement when aligned with *At*SHT and *At*HCT structures (Supplementary Figures 8B,C). As the loop is located near the active center, its movement might lead the active center open/close to the outside environment, which thus may help in maintaining the catalytic environment when it is in a close state or releasing the products when it is an open state. Since its proposed function is like a lid, we here named this loop as “lid-loop.”

These results are fully consistent with the interactions revealed from the modeled structures and suggest that key interactions from the proposed spermidine-binding pockets of *At*SDT and *At*SHT are critical for acyl acceptor substrate recognition and binding.

### Comparison on Spermidine Binding Pockets of *At*SDT and *At*SHT

Guided by structural information of *At*SDT and *At*SHT, we performed structure-based sequence alignments by using *At*SHT, *At*SDT, and other representative BAHD-transferase family homologues (Supplementary Figure 4) and found that residues in the proposed acyl-acceptor spermidine-binding pocket of *At*SDT were conserved with SDT homologues and were variable from SHT homologues (Supplementary Figure 4, shown as blue stars). Meanwhile, the residues from the *At*SHT acyl-acceptor spermidine-binding pocket were conserved among SHT homologues and were variable in SDT homologues (Supplementary Figure 4, shown as green circles). These findings further confirmed that different evolution strategies may be adopted for acyl acceptor spermidine binding in *At*SHT and *At*SDT.

**Figure 4:**
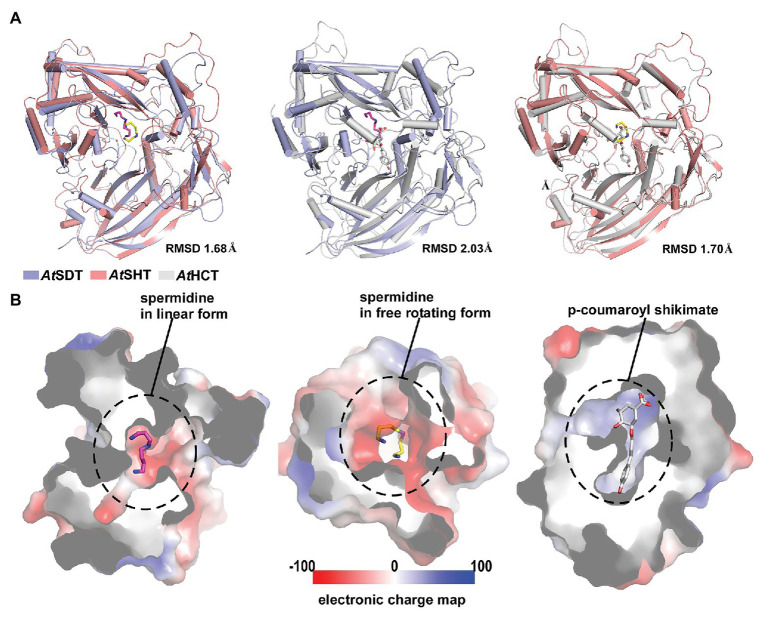
Overall structure and acyl-acceptor binding site comparison between *At*SDT, *At*SHT, and *At*HCT. **(A)** Structure superimpositions of *At*SDT and *At*SHT (left), *At*SDT, and *At*SHCT (middle), and *At*SHT and *At*HCT(right). Structures of *At*SDT, *At*SHT, and *At*HCT are shown with ribbons colored slate-blue, pink, and gray, respectively. Their respective acyl-acceptors are shown as stick models colored magenta, yellow, and gray. **(B)** Electron static surface models of *At*SDT(left), *At*SHT(middle) and *At*HCT(right) show the charge distributions in their acyl-acceptor binding sites.

In addition, we also compared the proposed acyl-acceptor binding pocket of *At*SHT and *At*SDT with that of *At*HCT. Although the overall structures of *At*SHT and *At*SDT are very similar to that of *At*HCT (the RMSDs of *At*HCT with *At*SHT and *At*SDT are 1.70 Å and 2.03 Å, respectively; Figure 4A), the interior features and the electrostatic potential of the acyl acceptor binding pockets of *At*SHT and *At*SDT differ significantly from those of *At*HCT. The formers are composed of negatively-charged residues to accommodate spermidine, while the latter is mainly comprised of positively-charged residues suitable for attracting and binding shikimate (Figure 4B). This suggests that it may be possible for us to predict and evaluate the acyl-acceptor substrate preference of unknown BAHD transferase family proteins based on the charge distributions of the residues comprising the acyl acceptor binding pocket.

### Prediction of Unknown BAHD Family Proteins

**Figure 5:**
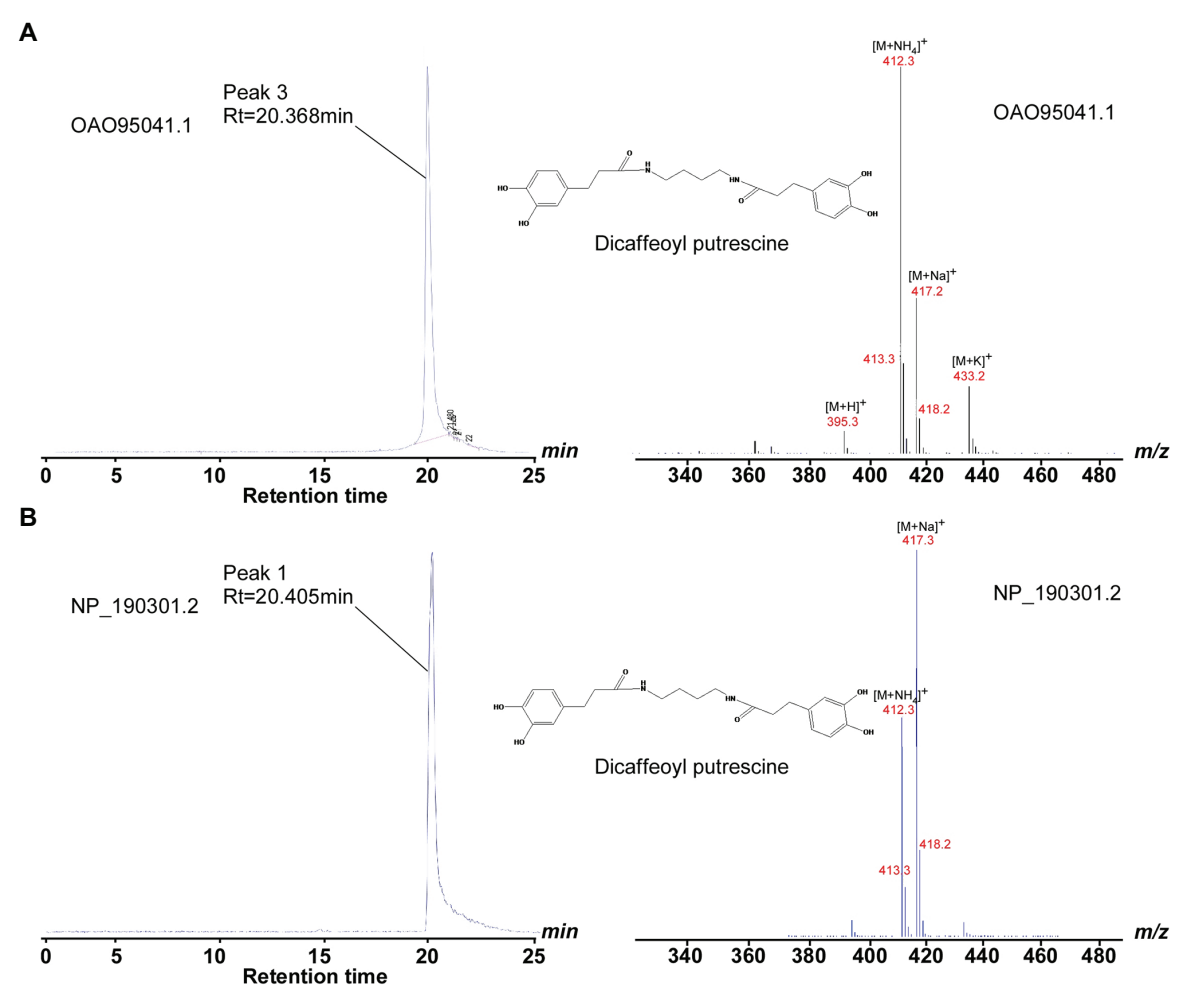
HPLC and LC/MS profiles show the catalytic activity of OAO95042.1 and NP_190301.2. **(A)** Left panel, HPLC profile of methanolic extracts showing products of wild-type OAO95042.1 reaction with caffeoyl-CoA and putrescine substrates; right panel, LC/MS fragmentation of di-caffeoyl putrescine (SIM mode, m/z 412). **(B)** Left panel, HPLC profile of methanolic extracts showing products of wild-type NP_190301.2 reaction with caffeoyl CoA and putrescine substrates; right panel, LC/MS fragmentation of di-caffeoyl putrescine (SIM mode, m/z 412). The di-caffeoyl putrescine (MW 412) perhaps easily formed the dehydration product (MW 394) in the reaction or detection conditions, and the peaks of 395.3, 412.3, 417.3, and 433.2 are [M+ H]+, [M+ NH4]+, [M + Na]+, and [M + K]+ of its dehydration products, respectively.

**Figure 6:**
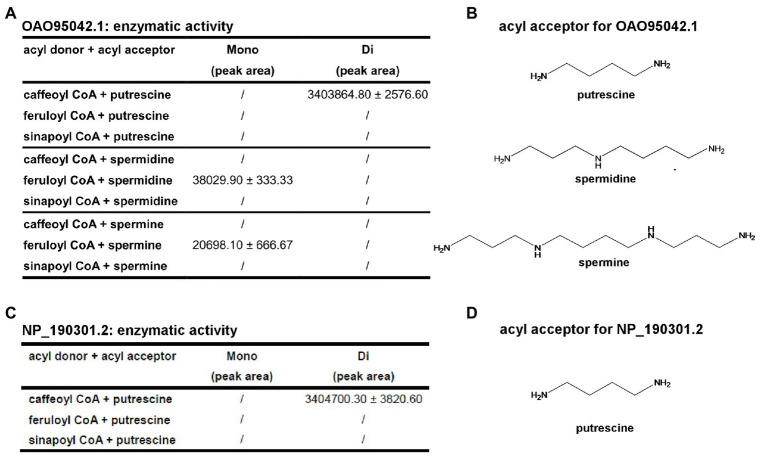
Summary of the catalytic activity of OAO95042.1 and NP_190301.2 with different acyl donors and acceptors. **(A)** Identification and quantification of products from different acyl acceptors and acyl donors for OAO95042.1(AT5G07080.1). **(B)** Acyl acceptors utilized by OAO95042.1. **(C)** Identification and quantification of products from different acyl acceptors and acyl donors for NP_190301.2(AT3G47170). **(D)** Acyl acceptor utilized by NP_190301.2. Values are means ± S.D. and error bars indicate the S.D. for three biological replicates.

With the structural information (especially the acyl-acceptor interaction residues’ distributions) from *At*SDT and *At*SHT, we expanded our analysis on other unknown BAHD-family proteins. A total number of 12,768 non-redundant protein sequences from the National Center for Biotechnology Information (NCBI) protein database was identified, including 49 *A. thaliana* BAHD transferase proteins (Dataset 1). Since the SSN can not only can display the same topology compared with phylogenetic tree (Davidson et al., 2018; Burroughs et al., 2019), but also have an advantage dealing with a large sequence dataset and a better global overview, we applied the SSN analysis in studying the BAHD-protein family sequences. The SSN map was generated using Pythoscape (Barber and Babbitt, 2012), where nodes represent sequences, and edges represent pairwise local alignments with e-values cut-off of 1e^-51.5^ (Supplementary Figure 9). Forty-nine *A. thaliana* BAHD transferase proteins were assigned to the SSN map where 18 functionally characterized sequences (Supplementary Table S1) were marked as purple triangle and 31 functionally unknown sequences were marked as blue circles (Supplementary Figure 9). The *At*SDT, *At*SCT, *At*SHT, and *At*HCT proteins were also highlighted in this map. Interestingly, *At*SDT and *At*SHT were present in two different clusters in the SSN map. The *At*SDT cluster contains 62 nodes, including 437 sequences in which *At*SCT, the spermidine dicoumaroyl transferase, is also present (sequence identity between *At*SDT and *At*SCT is 52.5%). The *At*SHT belongs to a more complicated cluster which contains several sub-clusters. The *At*SHT sub-cluster contains 96 nodes including 592 sequences and *Md*SHT, and *Ci*SHT are in the same node with *At*SHT. The sub-cluster containing hydroxycinnamoyl-CoA:shikimate hydroxycinnamoyl transferase (HCT) connected with *At*SHT sub-cluster showing the close relationship with *At*SHT. Indeed, the sequence of *At*HCT in this sub-cluster shows higher sequence identity with *At*SHT (sequence identity 36.4%) rather than with *At*SDT (sequence identity 22.2%) or *At*SCT (sequence identity 20.5%). To our surprise, there are two uncharacterized *A. thaliana* BAHD proteins (OAO95042.1 and NP_190301.2) in the same cluster with *At*SDT and *At*SCT. We then further evaluate their acyl-acceptor substrate preference on the basis of the charge distributions of the residues comprising the acyl-acceptor binding pocket. The structures of OAO95042.1 and NP_190301.2 were modeled using the *At*SDT structure as a template through the online Swiss-modeling program (Arnold et al., 2006), and negatively charged acyl-acceptor substrate binding pockets were found (Supplementary Figure 11), suggesting a basic substrate with characteristics similar to polyamines. For verification, the corresponding genes, *AT5G07080* and *AT3G47170* for OAO95042.1 and NP_190301.2, respectively, were cloned from *A. thaliana* and recombinant proteins were expressed in *E. coli*. The potential activity was tested using caffeoyl/feruloyl/sinapoyl-CoA as acyl donors and positively charged molecules putrescine/spermidine/spermine as acyl acceptors. The HPLC-MS results showed that both *AT5G07080* and *AT3G47170* encoded enzymes that could efficiently convert putrescine and caffeoyl-CoA to di-caffeoyl putrescine (Figures 5, 6 and Supplementary Figure 12). Further, OAO95042.1 enzyme can even convert spermidine/spermine and feruloyl CoA to mono-feruloyl spermidine/spermine (Figure 6 and Supplementary Figure 12). Results also suggested that the enzyme encoded by *AT5G07080* has a preference for feruloyl-CoA binding, but little acyl-acceptor specificity, while the enzyme encoded by *AT3G47170* has a preference for caffeoyl CoA and putrescine.

The successful prediction on substrate specificity of *AT5G07080*- and *AT3G47170* encoded BAHD enzymes demonstrated the feasibility of predicting and identifying possible substrates for uncharacterized BAHD-family transferases on the basis of the charge distributions of the residues comprising the acyl-acceptor binding pocket. It would provide great possibilities for us to gain a better understanding of the functionally uncharacterized BAHD-family proteins, and the potential strategy to predict substrates would be very useful for uncharacterized plant metabolite enzymes.

## Discussion

Phenolamides are ubiquitous secondary metabolites in plants. They are produced by BAHD-family acyltransferases that mono-conjugation of aromatic monoamines or poly-conjugation of aliphatic polyamines with phenolic acids. More and more research findings have highlighted the importance of phenolamides in diverse plant physiological processes, including defense responses and development. However, BAHD-family acyltransferases usually only share low sequence similarities among them, which makes it extremely challenging to predict the substrate specificity of uncharacterized BAHD family enzymes from their amino acid sequences. To solve this remaining question, our work here provides a new feasible methodology for the substrate specificity prediction of unknown BAHD family transferases in plants. The phylogenetic tree has been used for a long time on the function analysis of the unknown genes; however, with the limitation of gene numbers, the results are less reliable and irreproducible (Shen et al., 2020). By using the SSN, we could use all the information from the BAHD family sequences and have a global view on the classification of each gene, which would greatly accelerate the discovery of target genes. By combining the SSN analysis and structural information, we further clarified the potential target and validated them by more expensive *in vitro* experiment. Just based on this important finding, we successfully predicted the possible substrates of two uncharacterized BAHD-family enzyme OAO95042.1 and NP_190301.2, which turned out encoding the putrescine hydroxycinnamoyl transferases.

Our study also highlights the potential convergent-evolutionary way of *At*SDT and *At*SHT genes. Although both *At*SDT and *At*SHT use spermidine as an acyl-acceptor, the expression pattern and distribution of these two enzymes are different (Grienenberger et al., 2009; Luo et al., 2009). *At*SHT were mainly expressed in the tapetum of *Arabidopsis* anthers and synthiszedsynthesized fully substituted products. The other enzymes that synthesize the fully substituted products also have the similar expression pattern, such as *Ci*SHT, which promotes tetrahydroxycinnamoyl spermine accumulation in the pollen coat of the *Asteraceae* family, and *Md*SHT which synthesizes the trihydroxycinnamoyl spermidines in the pollen coat of core *Eudicotyledons*. Disrupting the function of *At*SHT would lead to abnormal formation of pollengrains in the *sht* mutant of *Arabidopsis*, indicating the probable function of trihydroxycinnamoyl spermidine derivatives in sporopollenin ultrastructure that the fully substituted products may provide for a barrier for pollen or may function as a supporting structure. Interestingly, according to our SSN map and phylogenetic tree, all of all these SHTs genes are close to HCTs, which are the key enzymes in lignin metabolism, and that both HCTs and SHTs may evolute from a same ancestor. On the other hand, the *At*SDT is mainly expressed in the seed and the root of *Arabidopsis* and synthesizes the mono- or di- substituted phenolamides. Unlike the SHT, the enzymes that synthesis the mono- or di- substituted phenolamides seem to be widely distributed in different organs and functions as plant biotic or abiotic stress responses. In our SSN map and phylogenetic tree, *At*SDT is far away from HCT, but close to our newly discovered putrescine transferases OAO95042.1 and NP_190301.2. Furthermore, according to our structures, residues in the proposed acyl-acceptor spermidine binding pocket of *At*SDT were conserved with SDT homologues and were variable from SHT homologues (Supplementary Figure 4, shown as blue stars). Meanwhile, the residues from the *At*SHT acyl acceptor spermidine-binding pocket were conserved among SHT homologues and were variable in SDT homologues (Supplementary Figure 4, shown as green circles). Taken together, we suggest that the *At*SHT and *At*SDT may undergo the convergent-evolutionary way and thus gain similar spermidine transferase activity.

The molecular mechanisms of multisite acylation of BAHD-family acyltransferases remain poorly understood so far. In this study, we tried to answer this tough question by determining the crystal structures of *At*SHT and *At*SDT, two BAHD family members catalyzing the multisite acylation of spermidine, and but showing different product profiles in *Arabidopsis thaliana*. We closely compared the differences in their potential spermidine binding pockets. The possible electron density shape for spermidine in the *At*SHT structure and our molecular docking results suggests that it may adopt a freely-rotating conformation in the center of the binding pocket (Supplementary Figure 13A) by interacting with residues Thr33, Asp314, Asp416, and His155, establishing equal probabilities for acylation of N^1^, N^5^, and N^10^ atoms in spermidine. In contrast, the possible electron density shape for spermidine in the *At*SDT structure, in combination with the molecular docking results, suggests a linear conformation at the center of the binding pocket (Supplementary Figure 13B). Therefore, spermidine in *At*SDT could only be docked into the binding pocket in two different orientations (“N^1^ to N^10^” or “N^10^ to N^1^”). In view of these spermidine conformation differences in *At*SHT and *At*SDT, we propose a “linear/rotation” model here, which may be able to clarify the potential mechanism of the different acylation activities of *At*SHT and *At*SDT (Supplementary Figure 14). The full acylation activity of *At*SHT is enabled by the “freely-rotating” conformation adopted by the acyl-acceptor spermidine in the binding pocket, while *At*SDT only binds in a linear conformation that is limited to the “head-tail” acylation (Supplementary Figure 14). That is, the acyl-acceptor spermidine adopts a free-rotating conformation in *At*SHT and can undergo mono-, di- or tri-acylation; while the spermidine molecule in *At*SDT adopts a linear conformation, which only allows mono- or di-acylation to take place. Our biochemical results all support this proposal that changing the spermidine binding pattern will decrease or abolish the production of fully acylated products, and thus match our proposed “linear/rotation” model (Supplementary Figure 14). Meanwhile, by superpositioning *At*SDT and *At*SHT structure with *At*HCT, we observe a potential movement of the “lid-loop,” which is located near the active center and may function in the maintenance of catalytic environments and the release of products (Supplementary Figure 8).

In summary, our extensive structural and biochemical analyses on *At*SDT and *At*SHT in this study provides an excellent starting point for predicting the biochemical functions of uncharacterized BAHD-family enzymes and understanding multisite acylation in BAHD-family enzymes. However, to further elucidate the potential molecular mechanism underlying the differing acylation activities of *At*SHT and *At*SDT, crystal structures of *At*SDT and *At*SHT in complex with its acyl donor and acceptor are still anticipated in the future.

## Data Availability Statement

The datasets presented in this study can be found in online repositories. The names of the repository/repositories and accession number(s) can be found at: http://www.wwpdb.org/, 6LPV and 6LPW.

## Author Contributions

CW, JL, and WL designed experiments. CW and JL performed the bulk of the experiments. MM contributed to protein expression, purification, and crystallization. ZL and WH contributed to enzymatic assay experiments. PZ, CW, and WL analyzed the data and wrote the manuscript. PZ conceived the project. All authors contributed to the article and approved the submitted version.

### Conflict of Interest

The authors declare that the research was conducted in the absence of any commercial or financial relationships that could be construed as a potential conflict of interest.
