# Supplementary material for: Structural and Biochemical Insights Into Two BAHD Acyltransferases (AtSHT and AtSDT) Involved in Phenolamide Biosynthesis
Source: Front Plant Sci. 2021 Jan 13;11:610118. doi: 10.3389/fpls.2020.610118 (PMC7838080; doi:10.3389/fpls.2020.610118)
Supplement: Supplementary file 1 [file Data_Sheet_1.pdf]

Supplementary materials

Structural and biochemical insights into two BAHD acyltransferases (*At*SHT and *At*SDT) involved in phenolamide biosynthesis

Chengyuan Wang[1,3,#], **Jianxu Li**[1,#], **Miaolian Ma**[1], **Zhaozhu Lin**[2], **Wenli Hu**[1], **Wei Lin** [2,*], **Peng Zhang**[2,*]

[1] National Key Laboratory of Plant Molecular Genetics, Center for Excellence in Molecular Plant Sciences, Shanghai Institute of Plant Physiology and Ecology, Chinese Academy of Sciences, Shanghai, China.

[2] Department of Microbiology and Immunology, School of Medicine & Holistic Integrative Medicine, Nanjing University of Chinese Medicine, Nanjing, China.

[3] Present address: Waksman Institute and Department of Chemistry, Rutgers University, Piscataway, NJ, 08854, USA.

*, Corresponding authors.

E-mail addresses: pengzhang01@sibs.ac.cn OR weilin@njucm.edu.cn

[#], These authors contributed equally to this work.

Table S1 18 BAHD family proteins which have been functionally characterized

**Protein ID**	**Publications**
NP_174189.1	(Vanholme et al., 2019)
NP_001322183.1	(Vanholme et al., 2019)
NP_568587.2	(Gou, Yu, & Liu, 2009; Molina, Li-Beisson, Beisson, Ohlrogge, & Pollard, 2009)
NP_194919.1	(Choi et al., 2013; Schneider et al., 2012; Zhu et al., 2013)
NP_564853.2	(Leshem et al., 2012)
NP_189609.1	(Taguchi et al., 2010)
NP_201517.1	(Zheng, Qualley, Fan, Dudareva, & Chen, 2009)
NP_197782.1	(Panikashvili, Shi, Schreiber, & Aharoni, 2009)
NP_199606.1	(Zhang & Xu, 2018)
NP_568561.4	(Taguchi et al., 2010)
NP_193275.1	(Wang et al., 2012)
NP_194182.1	(Haslam, Manas-Fernandez, Zhao, & Kunst, 2012)
NP_193120.1	(Haslam et al., 2015; Pascal et al., 2013)

References

Choi, S., Cho, Y. H., Kim, K., Matsui, M., Son, S. H., Kim, S. K., . . . Hwang, I. (2013). BAT1, a putative acyltransferase, modulates brassinosteroid levels in Arabidopsis. *Plant Journal, 73*(3), 380-391. doi:10.1111/tpj.12036

Gou, J. Y., Yu, X. H., & Liu, C. J. (2009). A hydroxycinnamoyltransferase responsible for synthesizing suberin aromatics in Arabidopsis. *Proc Natl Acad Sci U S A, 106*(44), 18855-18860. doi:10.1073/pnas.0905555106

Haslam, T. M., Haslam, R., Thoraval, D., Pascal, S., Delude, C., Domergue, F., . . . Joubes, J. (2015). ECERIFERUM2-LIKE Proteins Have Unique Biochemical and Physiological Functions in Very-Long-Chain Fatty Acid Elongation. *Plant Physiology, 167*(3), 682-+. doi:10.1104/pp.114.253195

Haslam, T. M., Manas-Fernandez, A., Zhao, L. F., & Kunst, L. (2012). Arabidopsis ECERIFERUM2 Is a Component of the Fatty Acid Elongation Machinery Required for Fatty Acid Extension to Exceptional Lengths. *Plant Physiology, 160*(3), 1164-1174. doi:10.1104/pp.112.201640

Leshem, Y., Johnson, C., Wuest, S. E., Song, X., Ngo, Q. A., Grossniklaus, U., & Sundaresan, V. (2012). Molecular characterization of the glauce mutant: a central cell-specific function is required for double fertilization in Arabidopsis. *Plant Cell, 24*(8), 3264-3277. doi:10.1105/tpc.112.096420

Molina, I., Li-Beisson, Y., Beisson, F., Ohlrogge, J. B., & Pollard, M. (2009). Identification of an Arabidopsis feruloyl-coenzyme A transferase required for suberin synthesis. *Plant Physiol, 151*(3), 1317-1328. doi:10.1104/pp.109.144907

Panikashvili, D., Shi, J. X., Schreiber, L., & Aharoni, A. (2009). The Arabidopsis DCR Encoding a Soluble BAHD Acyltransferase Is Required for Cutin Polyester Formation and Seed Hydration Properties. *Plant Physiology, 151*(4), 1773-1789. doi:10.1104/pp.109.143388

Pascal, S., Bernard, A., Sorel, M., Pervent, M., Vile, D., Haslam, R. P., . . . Joubes, J. (2013). The Arabidopsis cer26 mutant, like the cer2 mutant, is specifically affected in the very long chain fatty acid elongation process. *Plant Journal, 73*(5), 733-746. doi:10.1111/tpj.12060

Schneider, K., Breuer, C., Kawamura, A., Jikumaru, Y., Hanada, A., Fujioka, S., . . . Sugimoto, K. (2012). Arabidopsis PIZZA has the capacity to acylate brassinosteroids. *PLoS One, 7*(10), e46805. doi:10.1371/journal.pone.0046805

Taguchi, G., Ubukata, T., Nozue, H., Kobayashi, Y., Takahi, M., Yamamoto, H., & Hayashida, N. (2010). Malonylation is a key reaction in the metabolism of xenobiotic phenolic glucosides in Arabidopsis and tobacco. *Plant Journal, 63*(6), 1031-1041. doi:10.1111/j.1365-313X.2010.04298.x

Vanholme, R., Sundin, L., Seetso, K. C., Kim, H., Liu, X., Li, J., . . . Boerjan, W. (2019). COSY catalyses trans-cis isomerization and lactonization in the biosynthesis of coumarins. *Nat Plants, 5*(10), 1066-1075. doi:10.1038/s41477-019-0510-0

Wang, M. J., Liu, X. Y., Wang, R., Li, W. C., Rodermel, S., & Yu, F. (2012). Overexpression of a putative Arabidopsis BAHD acyltransferase causes dwarfism that can be rescued by brassinosteroid. *Journal of Experimental Botany, 63*(16), 5787-5801. doi:10.1093/jxb/ers227

Zhang, Z. Q., & Xu, L. P. (2018). Arabidopsis BRASSINOSTEROID INACTIVATOR2 is a typical BAHD acyltransferase involved in brassinosteroid homeostasis. *Journal of Experimental Botany, 69*(8), 1925-1941. doi:10.1093/jxb/ery057

Zheng, Z., Qualley, A., Fan, B., Dudareva, N., & Chen, Z. (2009). An important role of a BAHD acyl transferase-like protein in plant innate immunity. *Plant Journal, 57*(6), 1040-1053. doi:10.1111/j.1365-313X.2008.03747.x

Zhu, W., Wang, H., Fujioka, S., Zhou, T., Tian, H., Tian, W., & Wang, X. (2013). Homeostasis of brassinosteroids regulated by DRL1, a putative acyltransferase in Arabidopsis. *Mol Plant, 6*(2), 546-558. doi:10.1093/mp/sss144

A

***At*SHT: enzymatic activity**

acyl donor + acyl acceptor	Mono (peak area)	Di (peak area)	Tri (peak area)
caffeoyl CoA + spermidine	164935.00 ± 276.31	/	24440.10 ± 132.54
feruloyl CoA + spermidine	869806.26 ± 423.11	798638.57± 285.22	234434.77 ± 382.52
sinapoyl CoA + spermidine	2143476.00± 546.89	1570069.40±267.17	/

B

***At*SDT: enzymatic activity**

acyl donor + acyl acceptor	Mono (peak area)	Di (peak area)	Tri (peak area)
caffeoyl CoA + spermidine	/	141959.8±225.15	/
feruloyl CoA + spermidine	/	8725031.47±165.72	/
sinapoyl CoA + spermidine	67346.53 ± 335.73	6055065.07±192.56	/

Supplementary Figure 1. Summary of the catalytic activity of *At*SHT and *At*SDT with different acyl donors and spermidine.

(A) Identification and quantification of products from different acyl donors and spermidine for *At*SHT. **(B)** Identification and quantification of products from different acyl acceptors and acyl donors for *At*SDT. Values are means ± S.D. and error bars indicate the S.D. for three biological replicates.

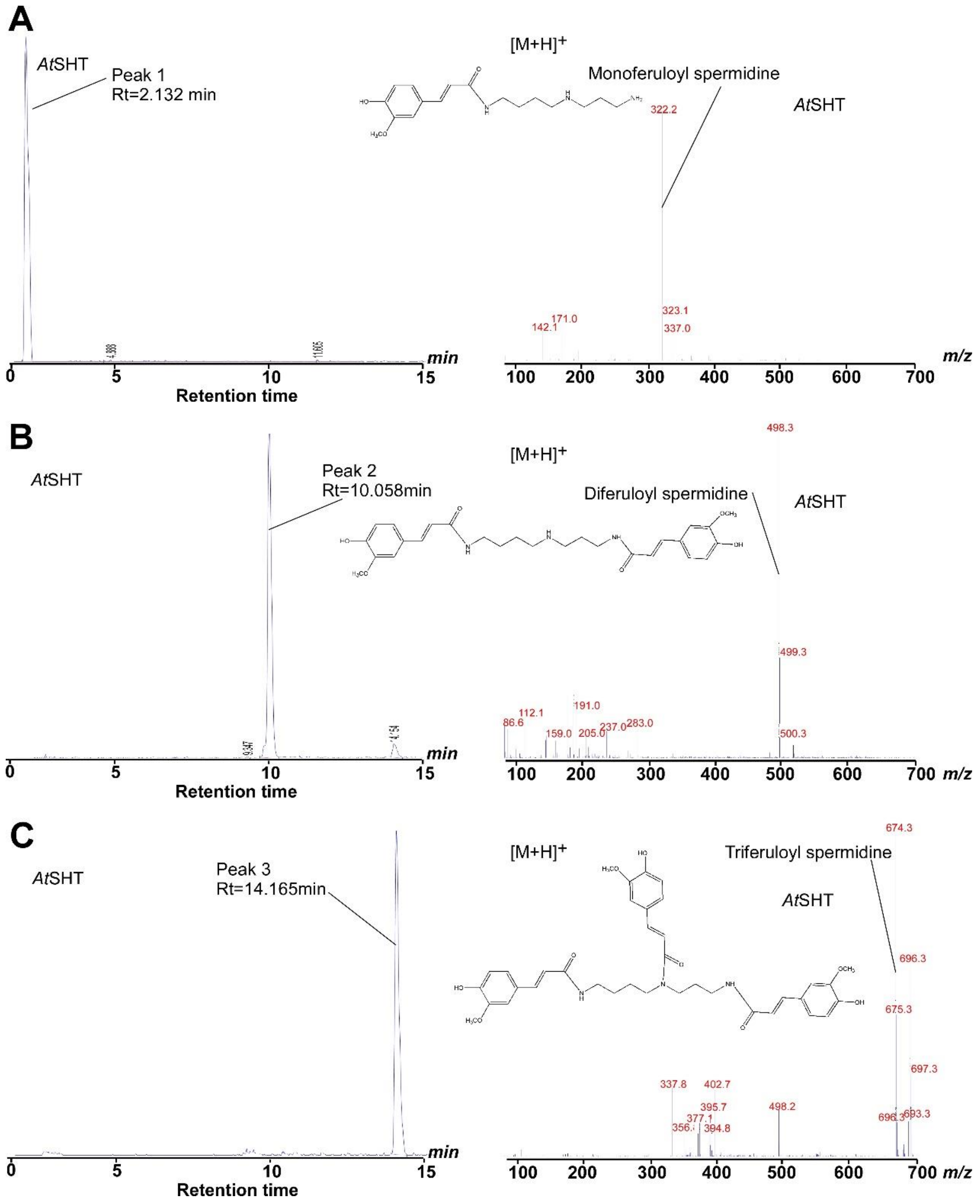

Supplementary Figure 2. Identification of reaction products of *At*SHT.

(A)-(C) Left panel, HPLC profiles of methanolic extracts showing products of *At*SHT reaction with feruloyl-CoA and spermidine substrates; Right panel, LC/MS fragmentation of mono-, di-, and tri-feruloyl-spermidine (SIM mode, m/z 322, 498 and 674, respectively).

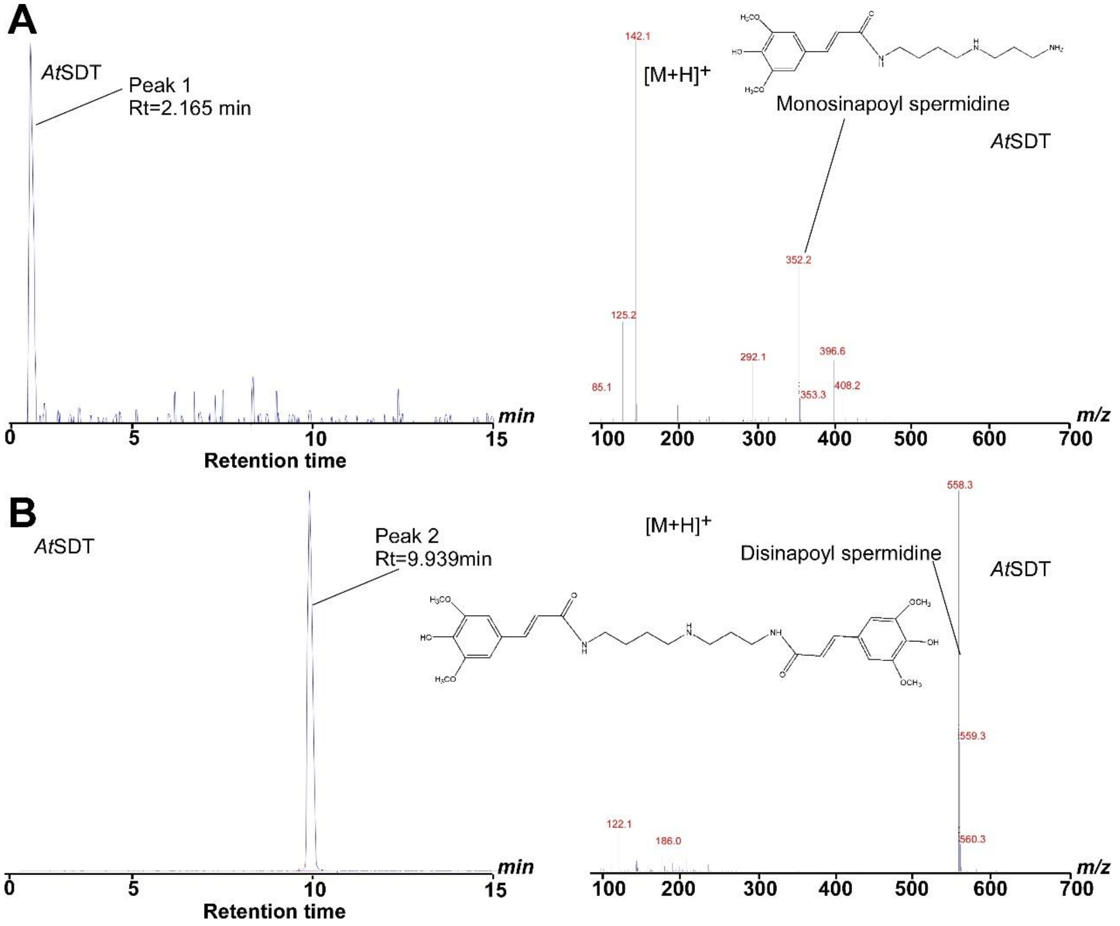

Supplementary Figure 3. Identification of reaction products of *At*SDT.

(A)-(B) Left panel, HPLC profiles of methanolic extracts showing products of *At*SDT reaction with sinapoyl-CoA and spermidine substrates; Right panel, LC/MS fragmentation of mono-, and di-sinapoyl spermidine (SIM mode, m/z 352 and 558, respectively).

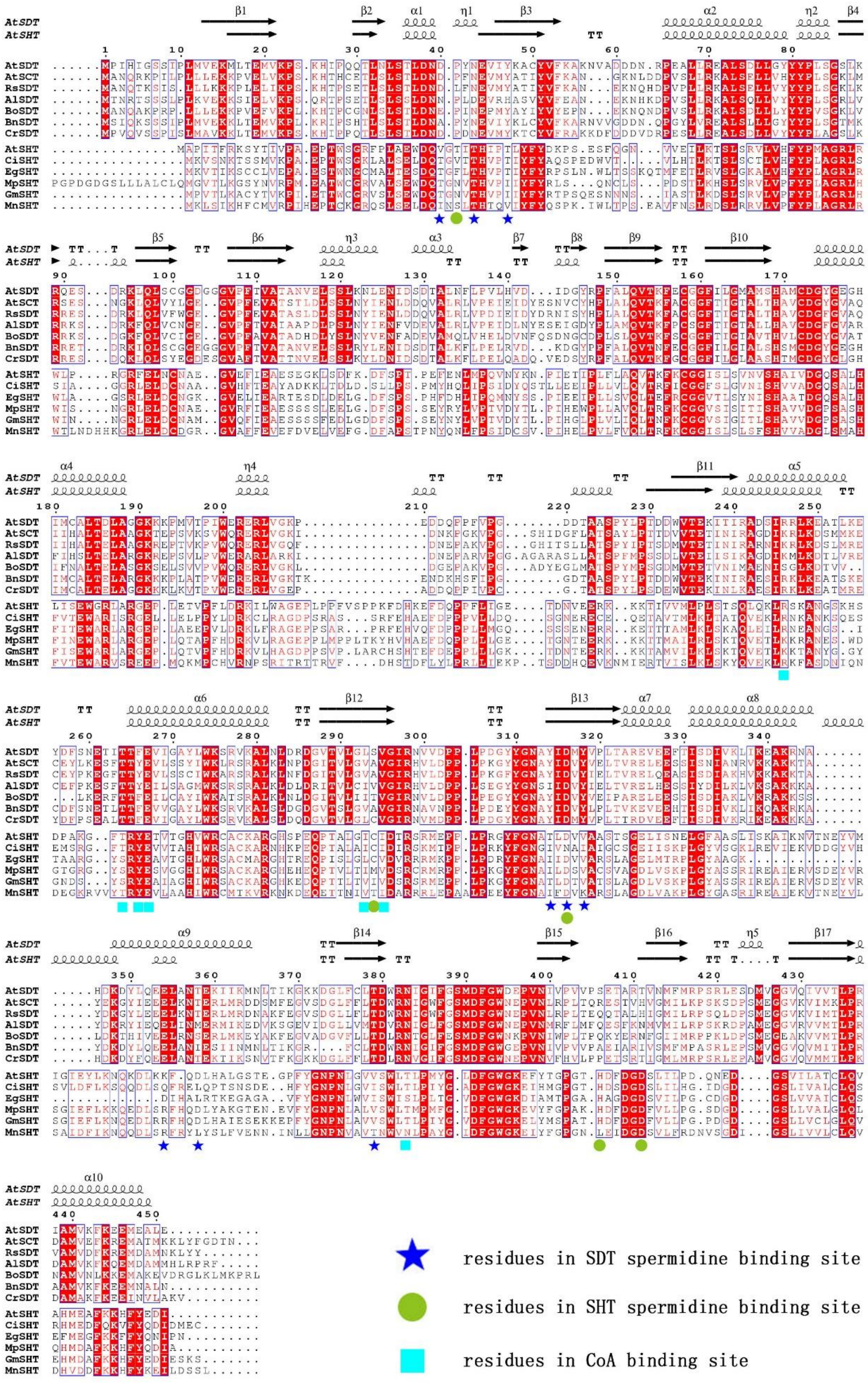
residues in SDT spermidine binding site
residues in SHT spermidine binding site
residues in CoA binding site

Supplementary Figure 4. Multiple-sequence alignment of different SDTs and SHTs.

The invariant residues among BAHD superfamily acyltransferases are highlighted in red, and conserved amino acids are boxed. residues in the spermidine binding site of *At*SDT are indicated with blue stars; residues in the spermidine binding site of *At*SHT are indicated with green cycles; residues in the CoA binding site of *At*SDT and *At*SHT are indicated with cyan squares. The secondary structure elements of *At*SDT and *At*SHT are shown at the top. *At*SDT, SDT from *Arabidopsis thaliana*; *At*SCT, SCT from *Arabidopsis thaliana*; *Rs*SDT, SDT from *Raphanus sativus*; *Al*SDT, SDT from *Arabidopsis lyrata*; *Bo*SDT, SDT from *Brassica oleracea*; *Bn*SDT, SDT from *Brassica napus*; *Cr*SDT, SDT from *Capsella rubella*; *At*SHT, SHT from *Arabidopsis thaliana*; *Ci*SHT, SHT from *Cichorium intybus*; *Eg*SHT, SHT from *Erythranthe guttata*; *Mp*SHT, SHT from *Mucuna pruriens*; *Gm*SHT, SHT from *Glycine max*; *Mn*SHT, SHT from *Morus notabilis*; The residue numbering is according to *At*SDT.

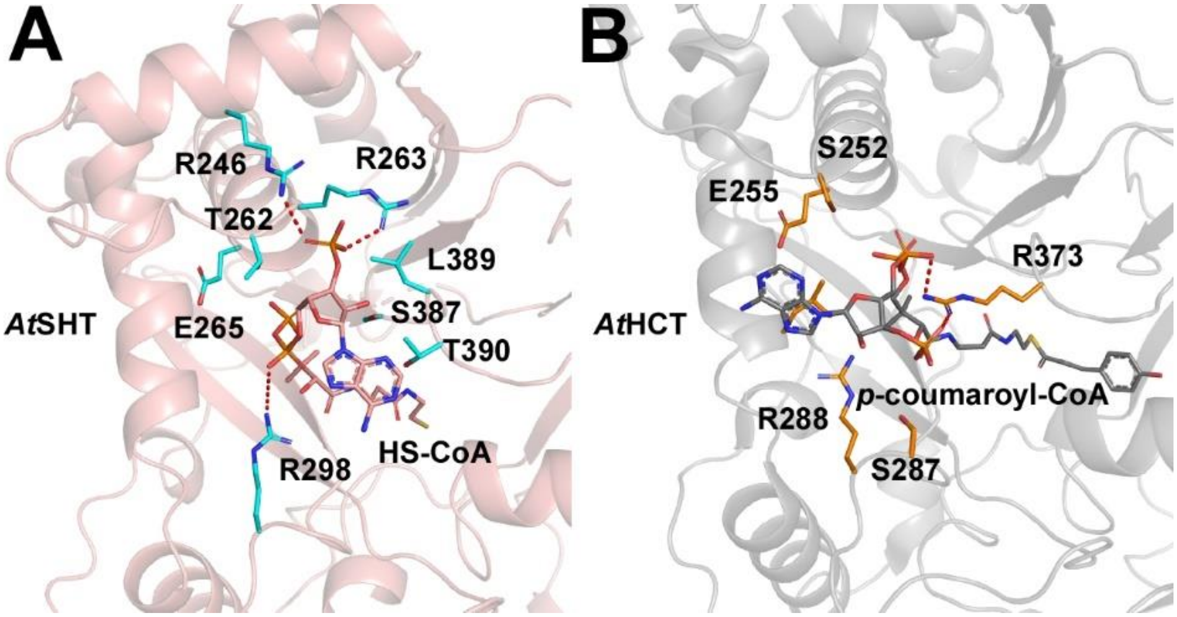

Supplementary Figure 5. Different conformations of CoA among BAHD family acyltransferases. comparisons of the adenylyl tail of HS-CoA among *At*SHT, *At*HCT, DmAT and TRI101.

(A) Interactions of the HS-CoA adenylyl group with surrounding residues (shown with side chains colored cyan) in *At*SHT. CoA-HS is shown as a pink stick model. **(B)** Interactions of the *p*-coumaroyl-CoA adenylyl group with surrounding residues (shown with side chains colored orange) in *At*HCT. *p*-coumaroyl-CoA is shown as a gray stick model.

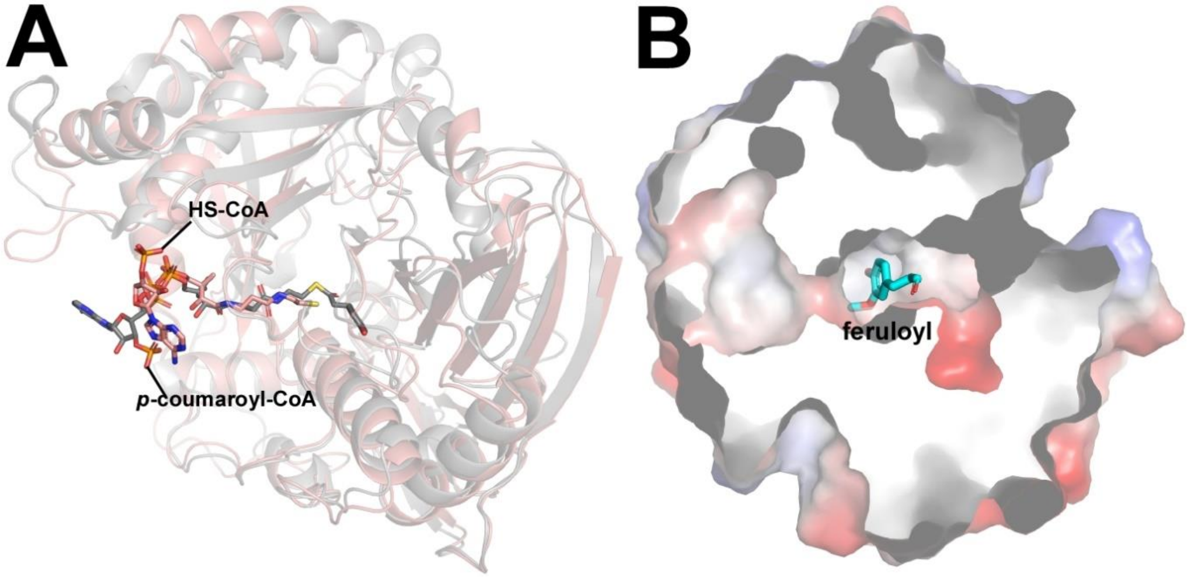

Supplementary Figure 6. Interactions of the feruloyl group of feruloyl-CoA in *At*SHT-CoA complex structure model.

The *At*SHT structure is shown with an electron static surface model, and the feruloyl group of feruloyl-CoA is shown as a cyan stick model. Other colors as in **Supplementary Figure 5**.

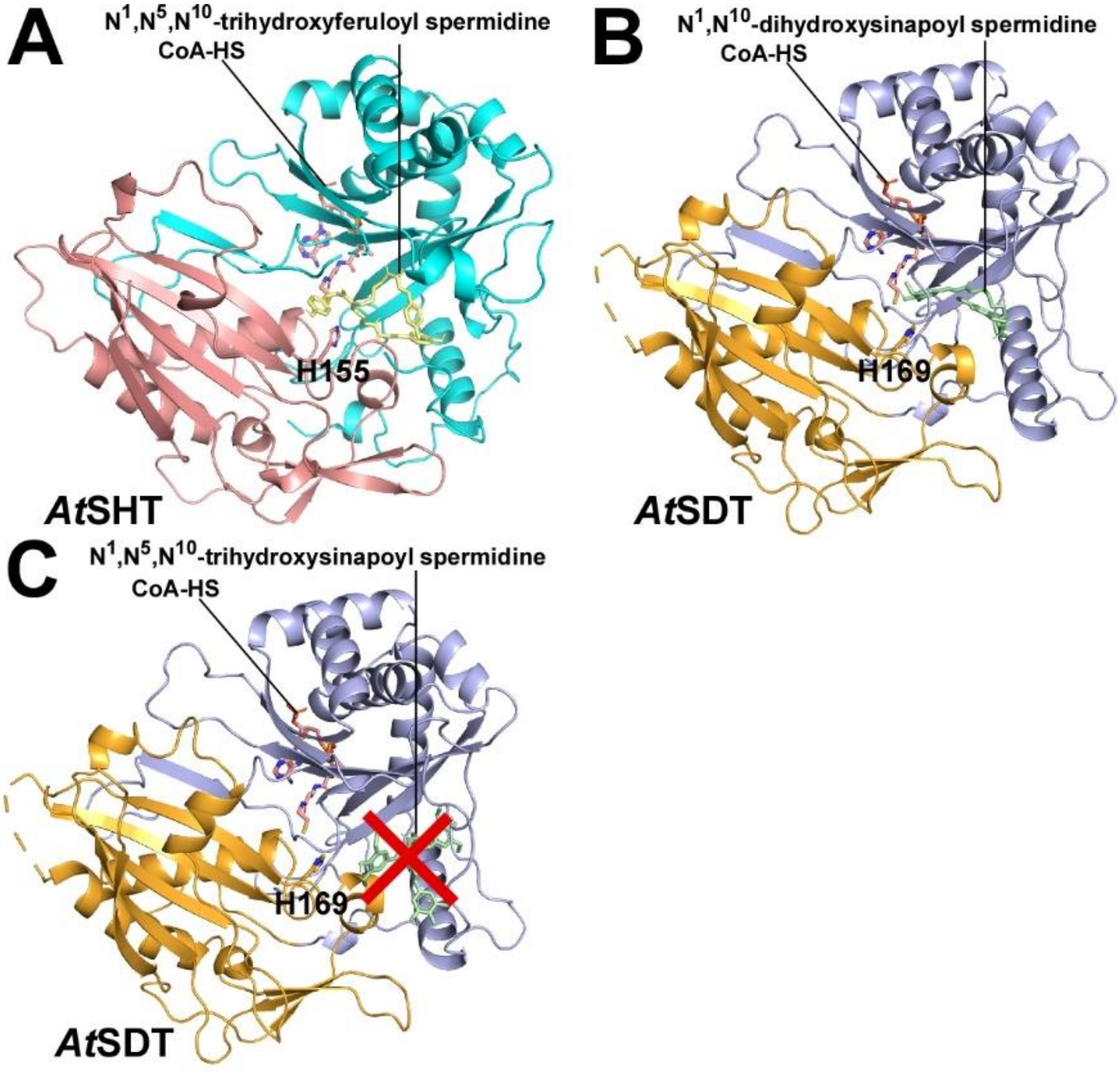

Supplementary Figure 7. Molecular docking studies of *At*SHT with CoA-HS and N^1,N^5,N^{10}-trihydroxyferuloyl spermidine, *At*SDT with CoA-HS and N^1,N^{10}-dihydroxysinapoyl spermidine, N^1,N^5,N^{10}-dihydroxysinapoyl spermidine. CoA-HS is shown with pink sticks; N^1,N^5,N^{10}-trihydroxyferuloyl spermidine is shown with yellow sticks, N^1,N^{10}-dihydroxysinapoyl spermidine and N^1,N^5,N^{10}-dihydroxysinapoyl spermidine are shown with palegreen sticks. Other Colors as in **Figure 2** and **3**.

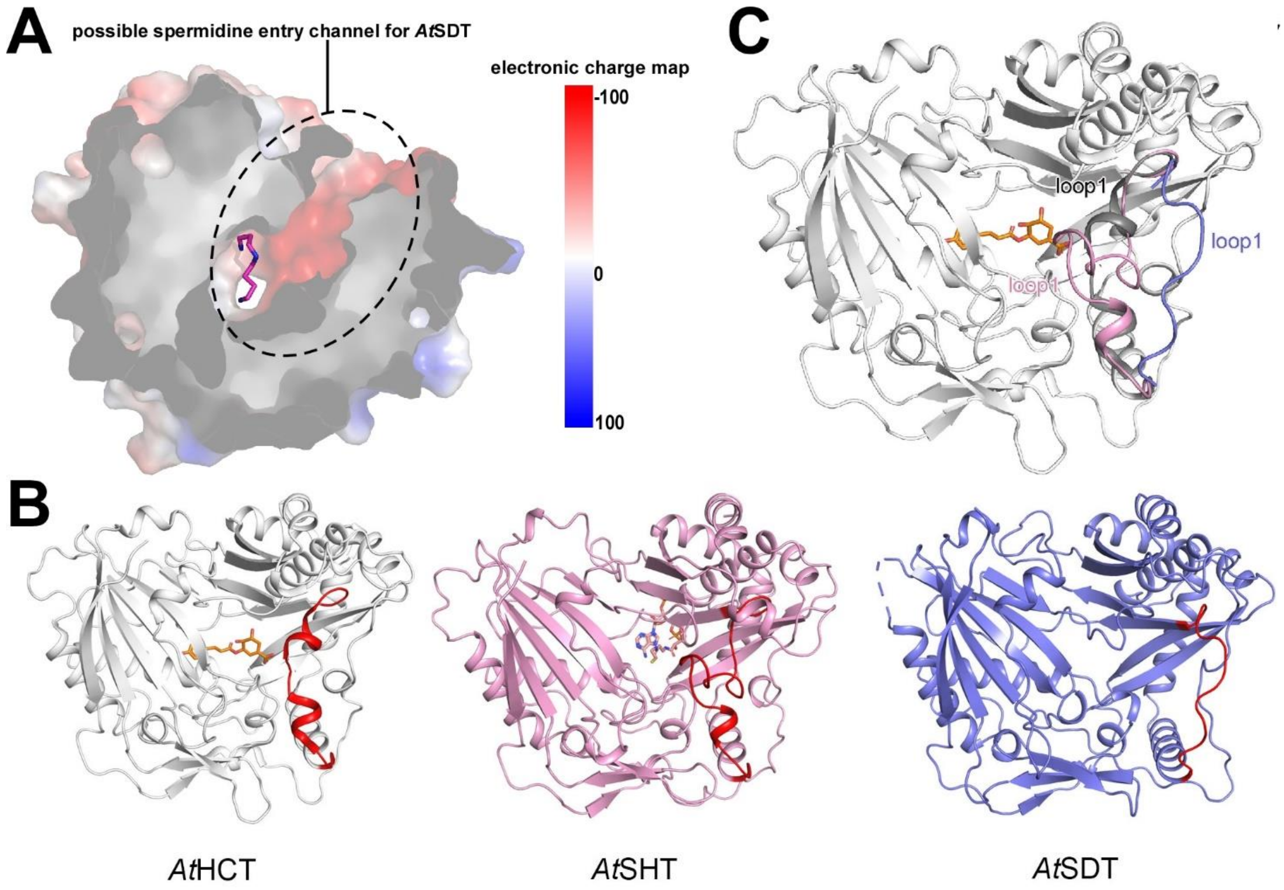

Supplementary Figure 8. (A) Possible spermidine entry channel of *At*SDT. Electron static surface model demonstrates the possible spermidine entry channel; **(B)** Overall crystal structure of *At*HCT, *At*SHT and *At*SDT colored in gray, pink and blue, respectively, the 'lid-loop" is colored in red; **(C)** Structure superimpositions of the 'lid-loop" from *At*HCT, *At*SHT and *At*SDT, colored in dark gray, pink and blue, respectively.

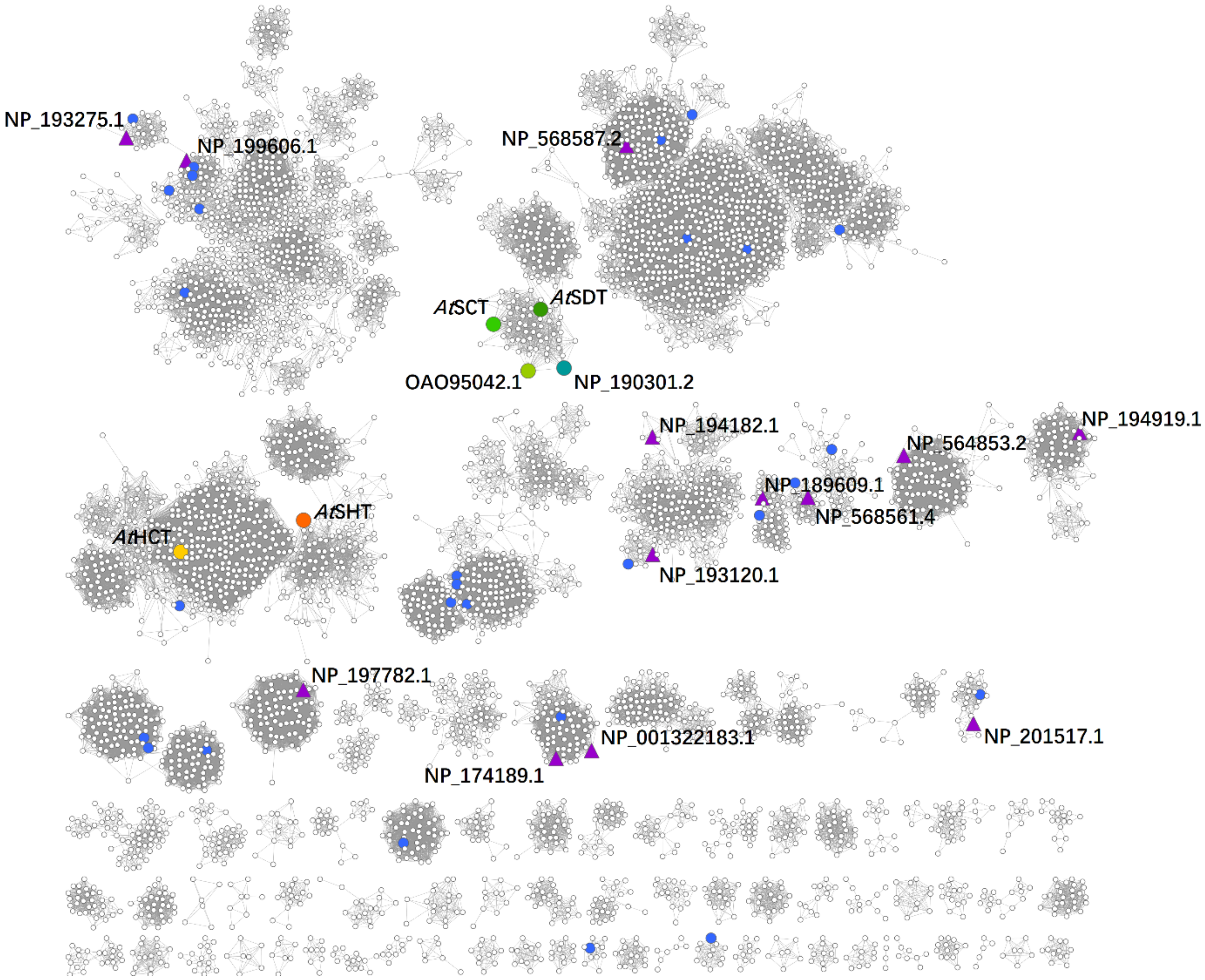

Supplementary Figure 9. BAHD transferase family similarity network map

Sequence similarity network of the BAHD transferase family with BLAST e-value cutoff set to $1x10^{-51.5}$. *At*SDT, *At*SCT, *At*SHT, *At*HCT, OAO95042.1, and NP_190301.2 are marked as large circle nodes colored dark-green, light-green, orange, yellow, lemon-green, and cyan, respectively. Functionally characterized BAHD family proteins in *Arabidopsis thaliana* (NP_193275.1, NP_199606.1, NP_568587.2, NP_194182.1, NP_194919.1, NP_564853.2, NP_189609.1, NP_568561.4, NP_193120.1, NP_197782.1, NP_174189.1, NP_001322183.1 and NP_201517.1) are marked as purple triangle nodes. 31 functionally unknown BAHD family proteins from *Arabidopsis thaliana* are marked as blue circles.

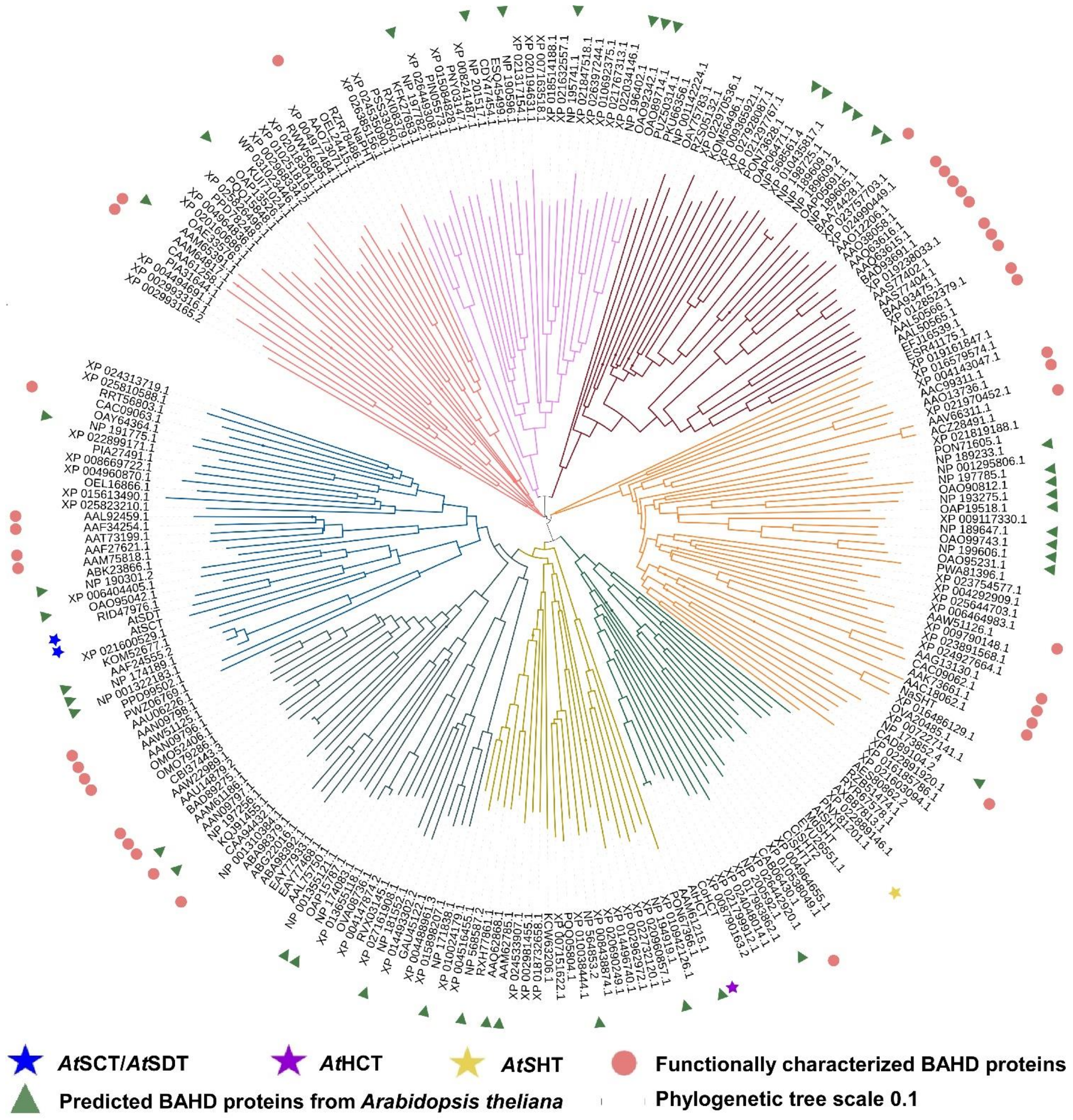

Supplementary Figure 10. Phylogenetic tree analysis of BAHD transferase family proteins.

The evolutionary history was inferred using the Neighbor-Joining method. The tree is drawn to scale, with branch lengths in the same units as those of the evolutionary distances used to infer the phylogenetic tree. The evolutionary distances were computed using the Poisson correction method and are in the units of the number of amino acid substitutions per site. This analysis involved 227 amino acid sequences. All ambiguous positions were removed for each sequence pair (pairwise deletion option). There were a total of 1608 positions in the final dataset. Evolutionary analyses were conducted in MEGA X. The phylogenetic tree includes 8 clades which are colored in red, pink, dark red, orange, green, yellow, dark green and light blue; *At*SCT and *At*SDT or *At*HCT or *At*SHT are labelled using

five-pointed star and colored in blue, purple and orange, respectively; functional characterized BAHD protein and predicted BAHD proteins from *Arabidopsis thaliana* are labelled using red circle and green triangle, respectively.

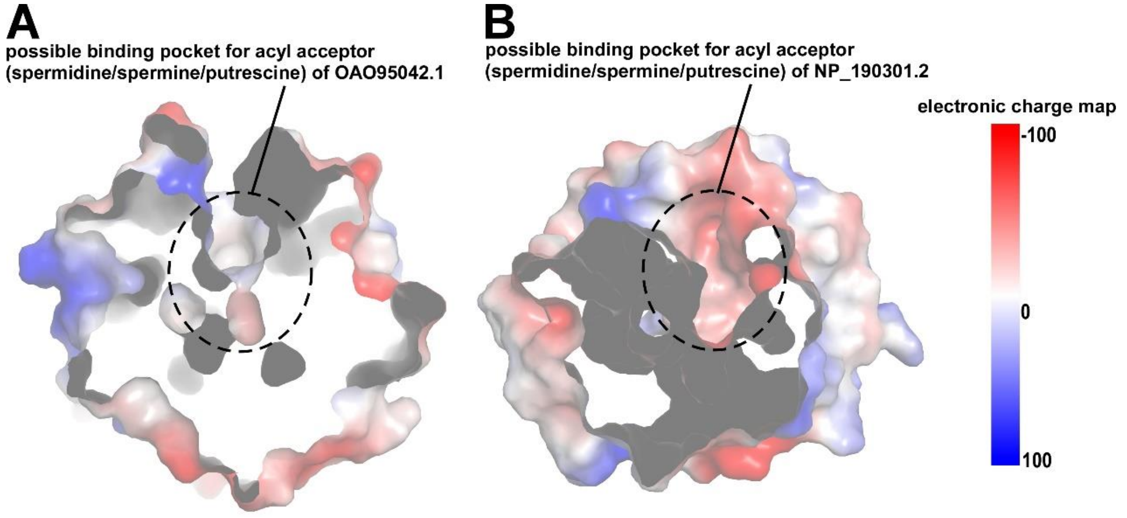

Supplementary Figure 11. Possible acyl acceptor binding sites of OAO95042.1 and NP_190301.2. The structures of OAO95042 **(A)** and NP_190301.2 **(B)** were generated using *At*SDT structure as a template.

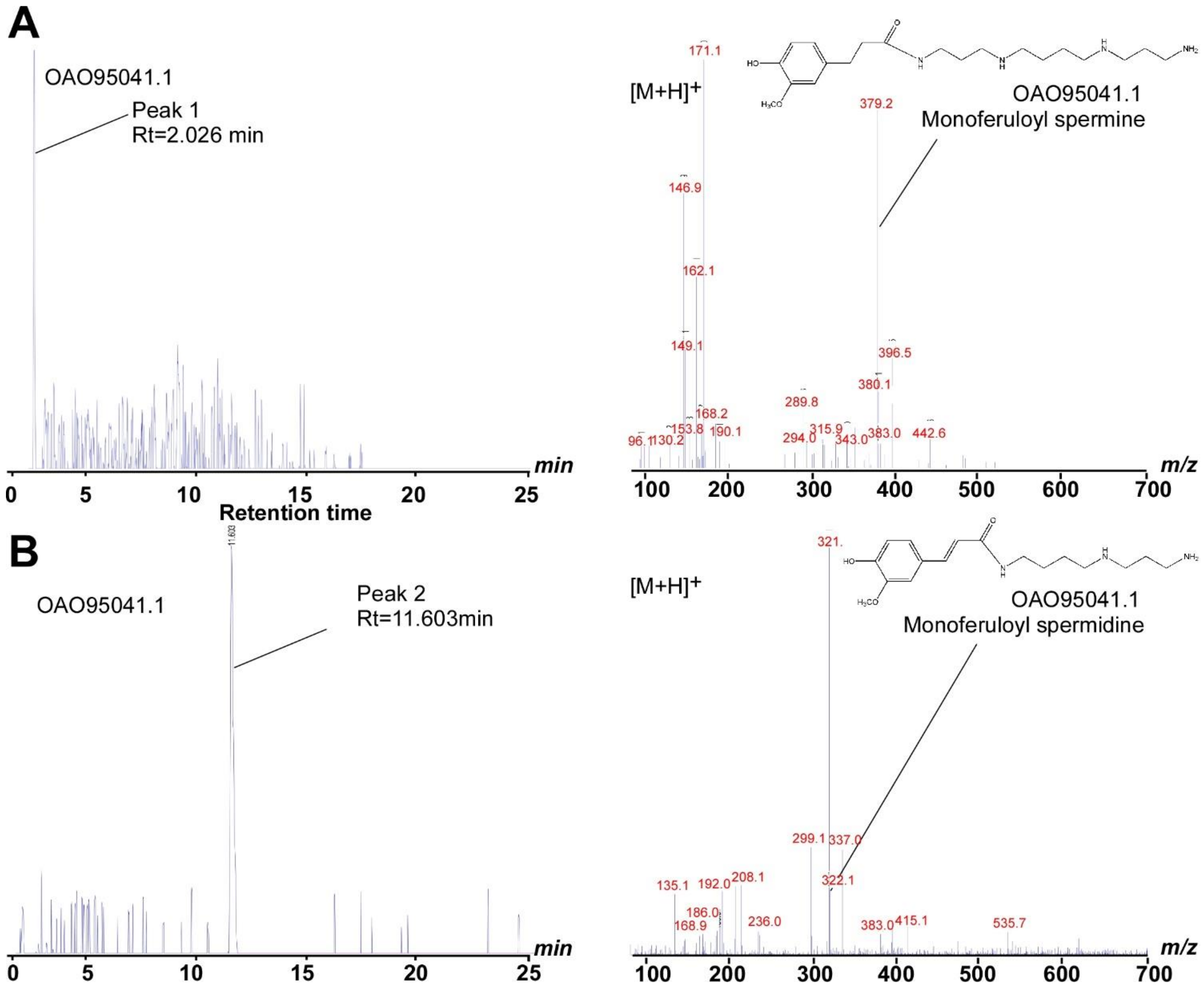

Supplementary Figure 12. HPLC and LC/MS profiles show the catalytic activity of OAO95042.1 and NP_190301.2.

(A) Left panel, HPLC profiles of the peak-1 of OAO95042.1 reaction product with ferulpoyl-CoA and spermine substrates; Right panel, LC/MS fragmentation of mono-feruloyl spermine (SIM mode, m/z 379). **(B)** Left panel, HPLC profiles of the peak-2 of OAO95042.1 reaction product with feruloyl-CoA and spermidine substrates; Right panel, LC/MS fragmentation of mono-feruloyl spermidine (SIM mode, m/z 322).

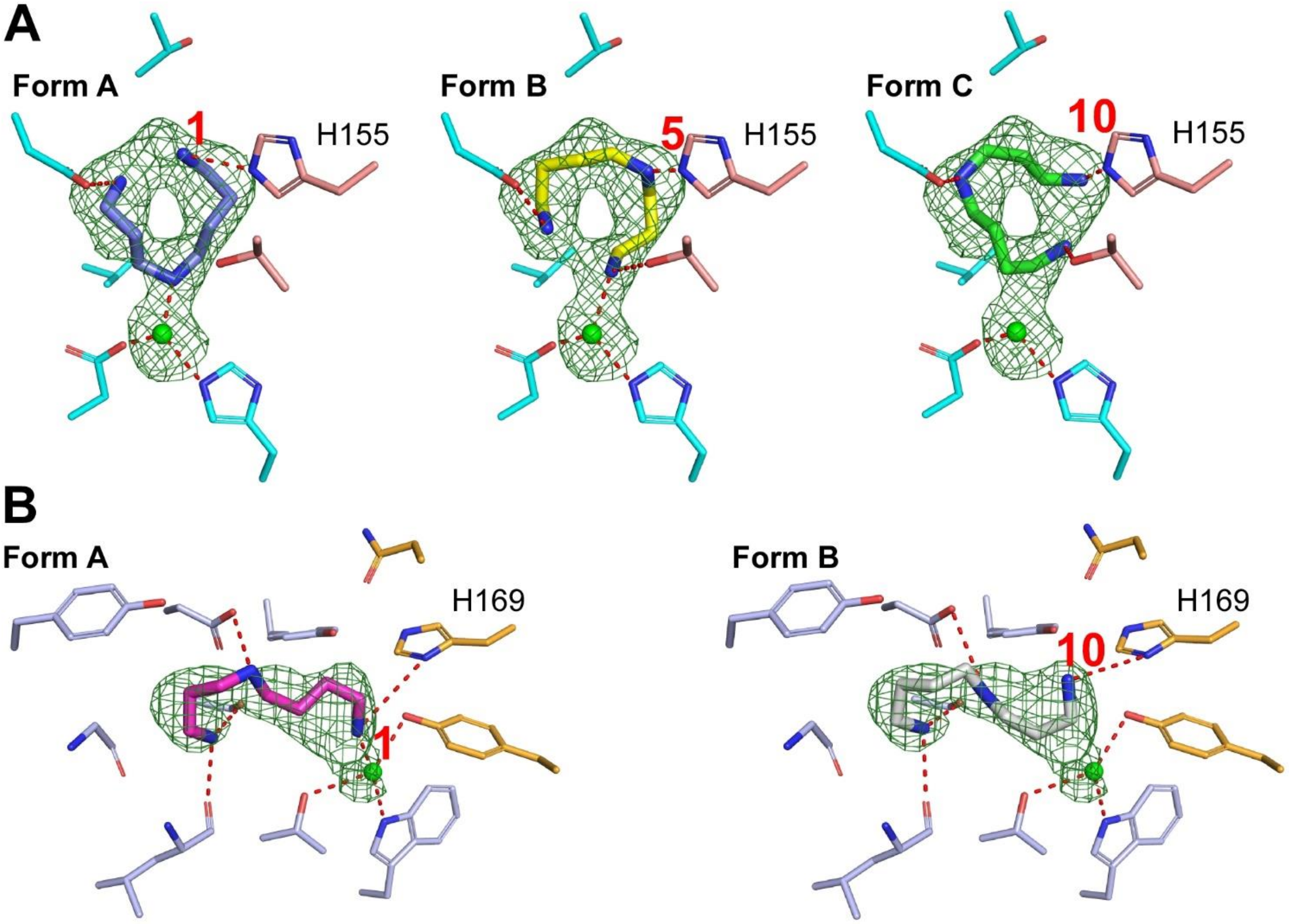

Supplementary Figure 13. The proposed acyl acceptor-spermidine-binding site of *At*SHT and *At*SDT.

(A) Three different conformations fit the possible Fo-Fc electronic density map (contoured at 2.5σ) for acyl-acceptor spermidine. The spermidine conformations are shown with stick models colored yellow, green, and blue. **(B)** Two different conformations of spermidine fit the possible Fo-Fc electronic density map (contoured at 2.5σ) for acyl-acceptor spermidine. The spermidine confirmations are shown with stick models colored magenta and gray.

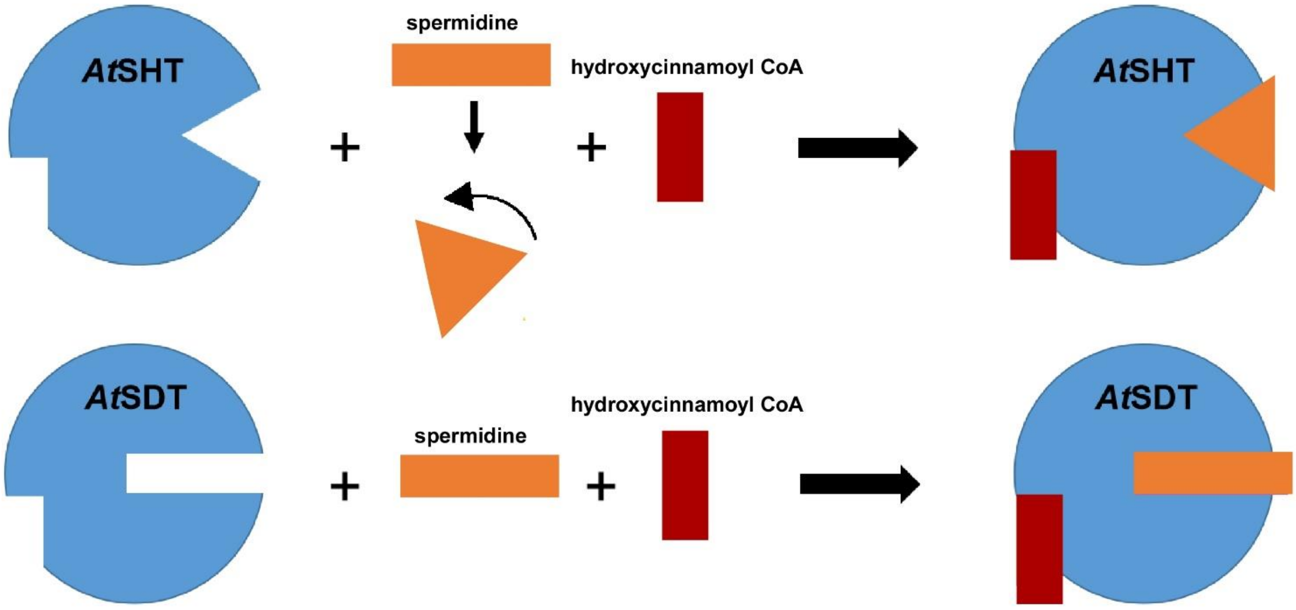

Supplementary Figure 14. Proposed multisite acylation models of *At*SHT and *At*SDT.

Proposed “linear-rotation” model for the multisite acylation mechanism of *At*SHT and *At*SDT. *At*SHT and *At*SDT are shown as blue circles; the hydroxycinnamoyl CoA is shown as a brown rectangle; Spermidine is shown as an orange triangle with *At*SHT and as an orange rectangle with *At*SDT.
